# Circ_0006174 Drives Triple Negative Breast Cancer Progression and Immune Escape Through Regulating miR‐3139/PD‐L1 Axis

**DOI:** 10.1002/kjm2.70156

**Published:** 2025-12-24

**Authors:** Yong‐Zhe Tang, Li Yang, Ling Wang, Xiao‐Qing Zhang, Jie Wang

**Affiliations:** ^1^ Department of Breast, The International Peace Maternity and Child Health Hospital, School of Medicine Shanghai Jiao Tong University Shanghai China; ^2^ Shanghai Key Laboratory of Embryo Original Diseases Shanghai China; ^3^ Department of Pharmacy, The International Peace Maternity and Child Health Hospital, School of Medicine Shanghai Jiao Tong University Shanghai China

**Keywords:** circ_0006174, immune escape, miR‐3139, PD‐L1, triple negative breast cancer

## Abstract

Triple‐negative breast cancer (TNBC) is characterized as an aggressive malignancy with limited therapeutic options. The circular RNA circ_0006174 has been implicated in oncogenic processes across various cancers; however, its specific role in the pathogenesis of TNBC remains unclear. This study employed a range of in vitro assays, including cell counting kit‐8, transwell invasion, ELISA, flow cytometry, RNA pull‐down, dual‐luciferase reporter, RNA immunoprecipitation, and western blotting, to investigate the function of circ_0006174 in TNBC. In vivo validation was conducted using xenograft models, with HE staining and immunohistochemistry. The results showed that overexpression of circ_0006174 was associated with poor prognosis in TNBC patients. Silencing circ_0006174 genetically resulted in reduced proliferation, invasion, and PD‐L1‐mediated immune‐moderation in TNBC cells. At the molecular level, circ_0006174 was found to sequester miR‐3139, leading to the upregulation of PD‐L1. Furthermore, the regulation of circ_0006174 by METTL3 was shown to be dependent on m6A methylation, with the primary m6A methylation site identified at position 315. In vivo interference led to a reduction in tumor burden and the decreased expression of ki‐67 and PD‐L1, while increasing the levels of miR‐3139 and IFN‐γ. Therefore, circ_0006174, regulated by METTL3‐mediated m6A methylation, promoted the progression of TNBC through the miR‐3139/PD‐L1 axis. This study was the first to validate the circ_0006174 as a novel m6A‐modified circRNA as well as the circ_0006174/miR‐3139/PD‐L1 axis as a potential target for TNBC.

## 1 Introduction

Breast cancer (BC) remains the most frequently diagnosed malignancy and the leading cause of cancer‐related mortality among women globally, underscoring its significance as a major global health concern. In 2022, there were 2,308,897 new cases (11.6%) and 665,684 new deaths (6.9%) attributed to this disease worldwide [1]. In China, breast cancer exhibits the highest incidence and the second‐highest mortality rate among female‐specific cancers, with 357,000 new cases and 75,000 new deaths reported in 2022, reflecting an increasing epidemiological burden [1]. Among its subtypes, triple‐negative breast cancer (TNBC) is the most aggressive, constituting 10% to 15% of breast cancer cases. TNBC is characterized by poor prognosis, high recurrence rates, and a pronounced tendency for metastasis [2]. Current clinical management is primarily based on therapeutic strategies including endocrine therapy, radiation therapy, cytotoxic chemotherapy, radical surgery, and molecular‐targeted treatments [3]. However, challenges such as tumor heterogeneity, acquired treatment resistance, and dose‐limiting toxicities persist, significantly hindering therapeutic effectiveness and expediting disease progression [4]. Epidemiological data indicate a marked disparity in survival outcomes between advanced metastatic disease, where median overall survival typically ranges from 24 to 36 months, and localized disease, which boasts a 10‐year survival rate of 85% [5]. These persistent clinical limitations underscore the urgent need for the development of novel therapeutic strategies that offer improved safety profiles and enhanced biological specificity, thereby revealing critical deficiencies in current treatment paradigms.

Circular RNAs (circRNAs), a category of non‐coding RNAs (ncRNAs), are characterized by a high degree of evolutionary conservation and a covalently closed circular structure [6]. CircRNAs have been identified as playing a significant role in BC [7, 8, 9, 10]. Furthermore, circRNAs have been demonstrated to function as molecular sponges for microRNAs (miRNAs), thereby influencing gene expression through the competitive endogenous RNA (ceRNA) mechanism [11]. Recent studies have consistently demonstrated that circRNAs, through miRNA sponging, play a pivotal role in BC. For example, circ_0000190 sequesters miR‐301a, thereby inhibiting proliferation, mobility, epithelial‐mesenchymal transition, and invasion while promoting apoptosis through the upregulation of MEOX2 in TNBC [12]. CircGSK3 facilitates immune evasion and tumor progression in breast cancer by upregulating miR‐338‐3p through the sponging of miR‐338‐3p [13]. circ_0006174 circularizing from exons 3 and 4 of the *RAD23B* gene [NM_002874], 349 nucleotides in length, which has been reported to be upregulated in colorectal cancer tissues and cells [14, 15, 16, 17, 18]. The knockdown of circ_0006174 influences various aspects of colorectal cancer progression, including radiosensitivity, proliferation, apoptosis, migration, invasion, chemoresistance, and metastasis [14, 15, 16, 17, 18]. However, the role of circ_0006174 in TNBC remains unexplored.

Thus, this study aimed to elucidate the function of circ_0006174 in the progression of TNBC. We anticipate that our research will highlight the potential clinical applications for BC diagnosis and management.

## 2 Materials and Methods

### 2.1 In Silico Analysis of circ_0006174 Expression in BC

The GSE182471 microarray dataset was obtained from the GPL21825 074301 Arraystar Human CircRNA microarray V2 platform, accessible via the GEO database. This dataset comprises five pairs of BC tissue samples and adjacent nontumor tissue (ANT) samples collected from five BC patients. Similarly, the GSE165884 microarray dataset was retrieved from the same platform within the GEO database, encompassing four pairs of BC tissue samples and ANT samples from five BC patients. The expression level of circ_0006174 in both the GSE182471 and GSE165884 datasets was analyzed using the LIMMA package in the R programming language.

### 2.2 Tissue Specimen

Seventy paired TNBC specimens and adjacent non‐tumor tissues (ANT) were collected from patients diagnosed with TNBC at our medical center and were immediately snap‐frozen in liquid nitrogen for preservation. Inclusion criteria: (1) Patients met the diagnostic and treatment criteria for breast cancer [19], as confirmed by clinical symptoms, imaging, surgery, and pathology; (2) Patients had no concurrent malignancies and had not received neoadjuvant therapies including radiation, chemotherapy, or biological treatments prior to surgical intervention; (3) Patients did not exhibit any allergic reactions to the drugs used in the study; (4) Patients possessed complete clinical data. Exclusion criteria: (1) Patients exhibiting functional impairments in critical organs such as the liver, kidneys, or heart; (2) Patients diagnosed with mental illness; (3) Patients who were pregnant. Patients were followed for 60 months, and 42 patients were alive. Histologically normal control tissues were obtained from areas maintaining a minimum clearance of 5 cm from tumor margins. The study protocol was approved by the Ethics Review Board of our institution and complied with the ethical standards set forth in the Declaration of Helsinki. Informed consent was obtained from all participants through voluntarily signed consent forms following a comprehensive explanation of the research procedures.

### 2.3 Cell Culture

MCF‐10A human normal mammary epithelial cells (JNO‐H0358), along with MDA‐MB‐231 (JNO‐H0364), BT549 (JNO‐H0346), and MDA‐MB‐468 (JNO‐H0369) TNBC cell lines, were supplied by Jennio‐Bio (Guangdong, China). MCF‐10A and BT549 cells were cultured in DMEM/F12 medium (PM150312, Procell, Wuhan, China), while MDA‐MB‐468 and MDA‐MB‐231 cells were maintained in Leibovitz's L‐15 medium (PM151010, Procell) supplemented with 1% penicillin–streptomycin solution (PB180120, Procell) and 10% fetal bovine serum (FBS, 164210, Procell), under conditions of 5% carbon dioxide (CO_2_) at 37°C.

### 2.4 Cell Transfection and Treatment

Short‐hairpin RNAs (shRNAs) specifically targeting circ_0006174 (designated as sh‐circ_0006174#1, #2, and #3), along with the corresponding control (sh‐NC), were procured from GenePharma (Shanghai, China, Table S1). These shRNAs were subsequently packaged into recombinant lentiviruses to facilitate the knockdown of circ_0006174 expression. According to the manufacturer's protocol, the lentiviruses were combined with BT549 and MDA‐MB‐231 cells to establish stable cell lines. Additionally, miR‐3139 mimic and miR‐NC, as well as the inhibitor (anti‐miR‐3139) and anti‐NC, were from GenePharma and were transfected into these cell lines using Lipofectamine 3000 (L3000001, Invitrogen, Carlsbad, CA, USA) to modulate miR‐3139 expression (Table S1). Furthermore, pcDNA vector plasmids containing PD‐L1 sequences or pLO5‐ciR containing circ_0006174 sequences were introduced into MDA‐MB‐231 and BT549 cells via Lipofectamine 3000 to upregulate the expression levels of PD‐L1 and circ_0006174 (Table S1). Small interfering RNA (siRNA) targeting METTL3, also sourced from GenePharma, was transfected into MDA‐MB‐231 cells to achieve downregulation of METTL3 expression.

To investigate the role of circ_0006174 in immune escape, CD8^+^ T cells were activated using IL‐2 (100 IU/mL, P5115, Beyotime, Shanghai, China) and Human T‐Activator CD3/CD28 Dynabeads (11161D, Gibco, Rockville, MD, USA) for 2 weeks. Following the co‐culture of transfected MDA‐MB‐231 and BT549 cells with CD8^+^ T cells for a duration of 24 h, the concentrations of interferon‐γ (IFN‐γ) and interleukin‐2 (IL‐2) in the co‐culture media were quantified using enzyme‐linked immunosorbent assay (ELISA). Concurrently, the percentage of CD8^+^ T cells was determined via flow cytometry.

### 2.5 RT‐qPCR

Total RNA was extracted from BC tissues and cells utilizing the TRIzol reagent (15,596,026, Thermo Fisher Scientific, Waltham, MA, USA), and complementary DNA (cDNA) synthesis was conducted using the Bio‐Rad ScripTM cDNA Synthesis Kit (1,708,890, Bio‐Rad Laboratories Inc., Hercules, CA, USA) in accordance with the manufacturer's instructions. The amplification reactions were performed with the 2× SYBR Master mix (RR820A, Takara, Dalian, China) on the Bio‐Rad CFX Manager system (Bio‐Rad Laboratories Inc.), employing the following thermal cycling conditions: an initial denaturation at 94°C for 5 min, followed by 40 cycles of 10‐s denaturation at 94°C, 45‐s annealing at 58°C, and 1‐min extension at 72°C. The expression of circ_0006174 was quantified using the 2^−ΔΔCT^ method, with GAPDH serving as the normalization control. For the detection of miR‐3139, reverse transcription was conducted using Bulge‐Loop miRNA‐specific primers (RiboBio Co. Ltd., Guangzhou, China). This process involved incubation at 42°C for 60 min, followed by 70°C for 10 min. The subsequent amplification process included an initial activation step at 94°C for 10 min, followed by 40 cycles of a 5‐s denaturation at 94°C, a 15‐s annealing at 58°C, and a 20‐s extension at 70°C, with U6 utilized as the endogenous control. The primer sequences used in this study were exhibited in Table 1.

**TABLE 1:** The sequences of primers.

Name	Forward (5′‐3′)	Reverse (5′‐3′)
*circ_0006174*	GCAACTGACAGGCAAAATCC	AGTTGTGGTGGTGGAGGAAG
*RAD23B*	CTTCCTCCACCACCACAACT	GGTGTCTCTGCTGGCTTTTC
*GAPDH*	GGTGGTCTCCTCTGACTTCAACA	GTTGCTGTAGCCAAATTCGTTGT
*miR‐3139*	UAGGAGCUCAACAGAUGCCUGUU	CGCAGGAGCUUUUGUUAUGUGCC
*U6*	CTCGCTTCGGCAGCACA	AACGCTTCACGAATTTGCGT

### 2.6 RNase R Assay

Total RNA was extracted from BT549 and MDA‐MB‐231 cells using TRIzol reagent and subsequently treated with 3 U/mg RNase R (HY‐P700300, MedChemExpress, Monmouth Junction, NJ, USA) at 37°C for 15 min. The expression levels of circ_0006174 and linear RAD23B were assessed via RT‐qPCR assay.

### 2.7 Actinomycin D Assay

BT549 and MDA‐MB‐231 cells were cultured in 12‐well plates and treated with 5 μg/mL actinomycin D when the cell density reached 70% for the experimental procedures. Cells were collected at predetermined time points for further analysis. The expression levels of circ_0006174 and linear RAD23B were evaluated using the RT‐qPCR assay.

### 2.8 Nuclear‐Cytoplasmic Fractionation Assay

A total of 1 × 10^7^ MDA‐MB‐231 and BT549 cells were collected and treated with the PARIS Kit (AM1921, Invitrogen) to separate the nuclear and cytoplasmic RNA. Following the wash, RNA was collected and used for RT‐qPCR as the above‐mentioned descriptions.

### 2.9 Cell Counting Kit‐8 (CCK‐8) Assay

Cells were cultured in 96‐well plates at a density of 1 × 10^5^ cells per well. The CCK‐8 solution (40203ES76, YEASEN, Shanghai, China) was administered at a volume of 10 μL per well and incubated with the cells for 2 h at 37°C. Optical density values were subsequently recorded at a wavelength of 450 nm using a microplate reader (Thermo Fisher Scientific).

### 2.10 Transwell Assay

Following resuspension in Leibovitz's L‐15 medium (for MDA‐MB‐231 cells) or DMEM/F12 medium (for BT549 cells) without FBS, cells were seeded at a density of 5 × 10^4^ cells per well in the upper compartment of transwell inserts (3422, 8‐μm pores, New York, NY, USA). These invasion chambers were pre‐coated with Matrigel matrix (356,234, Solarbio) prior to experimentation. Medium containing 20% FBS was placed in the lower compartment. After 24 h of incubation at 37°C with 5% CO_2,_ the Matrigel was removed. The cells were fixed with 4% paraformaldehyde (P0099, Beyotime, Shanghai, China) and stained with 0.1% crystal violet (C0121, Beyotime). Cellular invasion was quantified using an inverted microscope (Olympus) by counting stained cells across five distinct microscopic fields.

### 2.11 ELISA

ELISA kits, specifically product numbers PI511 and PI580 from Beyotime, were employed to quantify the concentrations of IFN‐γ and IL‐2. Optical density readings at a wavelength of 450 nm were acquired using a microplate reader from Thermo Fisher Scientific.

### 2.12 Flow Cytometry

Co‐cultured CD8^+^ T cells were isolated, washed with phosphate‐buffered saline (PBS), reconstituted, and incubated on ice for 30 min in the absence of light. This process was conducted in the presence of mouse monoclonal antibodies against CD3 (14‐0037‐82, Invitrogen, Carlsbad, CA, USA), CD8 (12‐0088‐42, Invitrogen), and IFN‐γ (14‐7319‐81, Invitrogen). Subsequently, the cells were analyzed using the CellQuest software on a FACScan flow cytometer (BD Biosciences, NJ, USA). Viable cells were identified and gated based on forward and side scatter properties. CD8^+^ T cells and IFN‐γ‐expressing CD8^+^ T cells were then identified using antibodies against CD3, CD8, and IFN‐γ.

### 2.13 Western Blotting

Protein samples lysed with radioimmunoprecipitation assay (RIPA) buffer, containing 20 μg of protein per lane, were separated by 10% sodium dodecyl sulfate‐polyacrylamide gel electrophoresis (SDS‐PAGE) and subsequently transferred onto polyvinylidene difluoride (PVDF) membranes. Following a blocking step, the membranes were probed with rabbit monoclonal anti‐PD‐L1 (1:1000, ab205921, Abcam, Cambridge, UK), rabbit polyclonal anti‐METTL3 (1:5000, ab240595, Abcam), and rabbit polyclonal anti‐GAPDH (1:2500, ab9485, Abcam). HRP‐conjugated goat anti‐rabbit IgG H&L (1:10,000, ab6721, Abcam) was employed in conjunction with a chemiluminescent substrate for band visualization (ChemiDoc MP). Densitometric analysis was performed using Image‐Pro Plus 6.0 with GAPDH normalization.

### 2.14 Online Prediction

The Starbase database platform (https://starbase.sysu.edu.cn/starbase2/) was used to identify miRNAs targeted by circ_0006174, while the Targetscan database (https://www.targetscan.org/vert_80/) was utilized to identify miRNAs targeted by PD‐L1. Prediction of m6A sites on circ_0006174 was conducted using the SRAMP tool (http://www.cuilab.cn/sramp).

### 2.15 RNA Pull‐Down

RNA‐protein interaction analyses were carried out using the Pierce Magnetic RNA‐Protein Pull‐Down Kit (20,164, Thermo Fisher) according to standardized protocols. Initially, biotin‐labeled circ_0006174 probes were conjugated with magnetic beads and incubated at room temperature for 2 h. Subsequently, the mixtures were incubated with HCM lysates at 4°C overnight. Following magnetic bead elution, the bound miRNAs were quantified via RT‐qPCR analysis.

### 2.16 Methylated Immunoprecipitation (RIP)‐qPCR

Methylated RIP‐qPCR was performed using the RIP kit (17‐10499, Millipore, Bedford, MA, USA) in accordance with the manufacturer's instructions. Cell lysis of MCF10A, MDA‐MB‐231, and BT549 lines was performed using 1 mL of RIP lysis buffer. Following a 10‐min incubation period, 100 μL of the lysate was extracted and stored at −80°C. Subsequently, a rabbit polyclonal anti‐m6A antibody (1:50, ab151230, Abcam) or normal rabbit immunoglobulin G (IgG) (1:50, ab2410, Abcam) was combined with protein A/G microbeads and incubated for 2 h at 4°C. RNA analysis was then conducted using qRT‐PCR, adhering to previously established protocols.

### 2.17 RNA Immunoprecipitation (RIP) Assay

The RIP assay was executed utilizing the Magna RIP Kit (Millipore). For MDA‐MB‐231 and BT549 cell lysates prepared with RIP buffer, immunoprecipitation was performed with magnetic beads conjugated with either anti‐Ago2 or control IgG antibodies (12‐370, Millipore). Additionally, magnetic beads were pre‐coated with 5 mg of antibodies specific to METTL3, METTL14, ALKBH5, FTO, and corresponding IgG, in accordance with the manufacturer's instructions. These coated beads were subsequently incubated with MDA‐MB‐231 cell lysates and treated with proteinase K to degrade proteins. Upon complex formation, the complexes were precipitated and separated from the beads. The extracted RNAs were then purified for subsequent qPCR analysis. The enrichment level was ascertained by normalizing the input.

### 2.18 Luciferase Assay

Constructs of circ_0006174 or PD‐L1 in both wild‐type (WT) and mutant (MUT) forms were cloned into the pGL3‐Basic luciferase vector (Promega, Madison, WI, USA) to create luciferase reporter plasmids. Subsequently, these WT or MUT plasmids were co‐transfected into MDA‐MB‐231 and BT54 cells alongside either a miR‐NC or miR‐3139 mimic using Lipofectamine 3000. Luciferase activity was quantified using the Dual Luciferase Reporter Gene Assay Kit (RG027, Beyotime), according to the manufacturer's instructions, 48 h post‐transfection.

To evaluate the effect of m6A modification on circ_0006174, four plasmids containing partial sequences of circ_0006174 were engineered to include either mutant or wild‐type m6A sites (M1–M3). For the luciferase reporter assays, MDA‐MB‐231cells with reduced expression of METTL3 were cultured in 24‐well plates at a density of 1 × 10^5^ cells per well. The plasmids were transfected into the cells using Lipofectamine 3000 (Thermo Fisher Scientific). After 24 h, the luciferase activity was assessed using the Dual Luciferase Reporter Gene Assay Kit.

### 2.19 Animal Experiment

Four‐week‐old 7‐Hu‐PBL‐NSG mice were procured from Shanghai Model Organisms (Shanghai, China) and housed under specific pathogen‐free conditions with regulated temperature and a 12‐h light/dark cycle. Mice (*n* = 6 per group) were implanted with MDA‐MB‐231 xenografts (2 × 10^6^ cells) [20] in the left flank, using lentivirus‐packaged sh‐NC or sh‐circ_0006174. Terminal anesthesia was administered via exposure to excess isoflurane vapor (R510‐22, RWD, Guangdong) prior to necropsy. Tumor analysis at the terminal stage included volumetric measurement (0.5 × L × W^2^), weight, histopathological examination (H&E), and immunohistochemical analysis (ki‐67/PD‐L1/IFN‐γ/CD8). All experimental procedures were approved by the Animal Research Ethics Committee of our institution.

### 2.20 Hematoxylin and Eosin (H&E) Staining

Tumor specimens were preserved in a 4% paraformaldehyde solution, followed by a series of sequential ethanol dehydrations. The tissues were then embedded in paraffin blocks (YA0011, Solarbio) and sectioned into 5 μm slices. These sections underwent H&E staining (G1120, Solarbio) and were subsequently examined using an optical microscope (Olympus) in conjunction with Image‐Pro Plus 6.0 digital imaging software (Media Cybernetics, USA) for detailed histological evaluation.

### 2.21 Immunohistochemistry

Tissue sections were subjected to antigen retrieval using a sodium citrate buffer (pH 6.0, P0081, Beyotime) at 94°C for 15 min. Following this, sections were blocked with 1% BSA and incubated overnight at 4°C with rabbit polyclonal antibodies: anti‐ki‐67 (1:500, ab15580, Abcam), anti‐PD‐L1 (1:100, PA5‐20343, Invitrogen), anti‐IFN‐γ (1:100, PA5‐95560, Invitrogen), and anti‐CD8 (1:100, PA5‐88265, Invitrogen). Detection was performed using HRP‐conjugated goat‐anti‐rabbit IgG (1:200, ab288151, Abcam) and DAB substrate. Counterstained slides were visualized at 200× magnification.

### 2.22 Terminal Deoxynucleotidyl Transferase Deoxyuridine Triphosphate (dUTP) Nick End Labeling (TUNEL) Assays

Paraffin embedded sections were deparaffinized with xylene and rehydrated through a graded ethanol series, followed by treatment with proteinase K (P9460, Solarbio) for 30 min at room temperature. After three washes with PBS, the sections were treated with 2% H_2_O_2_ for 20 min at room temperature. Subsequently, the sections were washed three times with PBS and incubated with TUNEL reaction mixtures (C1090, Beyotime) at 37°C for 1 h. The sections were then incubated with 50 μL of Streptavidin‐HRP (A0305, Beyotime) for 30 min at room temperature and developed with 500 μL of DAB (P0202, Beyotime) for 30 min at room temperature. Finally, the sections were counterstained with hematoxylin (C0107, Beyotime) and imaged using a light microscope (Olympus). The percentage of apoptotic cells was determined by calculating the ratio of TUNEL‐positive cells to the total number of cells.

### 2.23 Statistical Analysis

The data were represented as mean ± standard deviation (SD) of triplicate measurements. Intergroup differences were evaluated using the Student's *t*‐test for comparisons between two groups, and analysis of variance (ANOVA) with Bonferroni correction for multiple group comparisons, utilizing SPSS version 20.0. Survival outcomes were analyzed using the Kaplan–Meier method, with log‐rank tests employed for comparative analysis. Association between circ_0006174 expression and clinical‐pathological parameters was assessed using the chi‐square test. A *p*‐value of less than 0.05 was considered statistically significant for all analyses.

## 3 Results

### 3.1 Circ_0006174 Was Highly Expressed in TNBC

**FIGURE 1:**
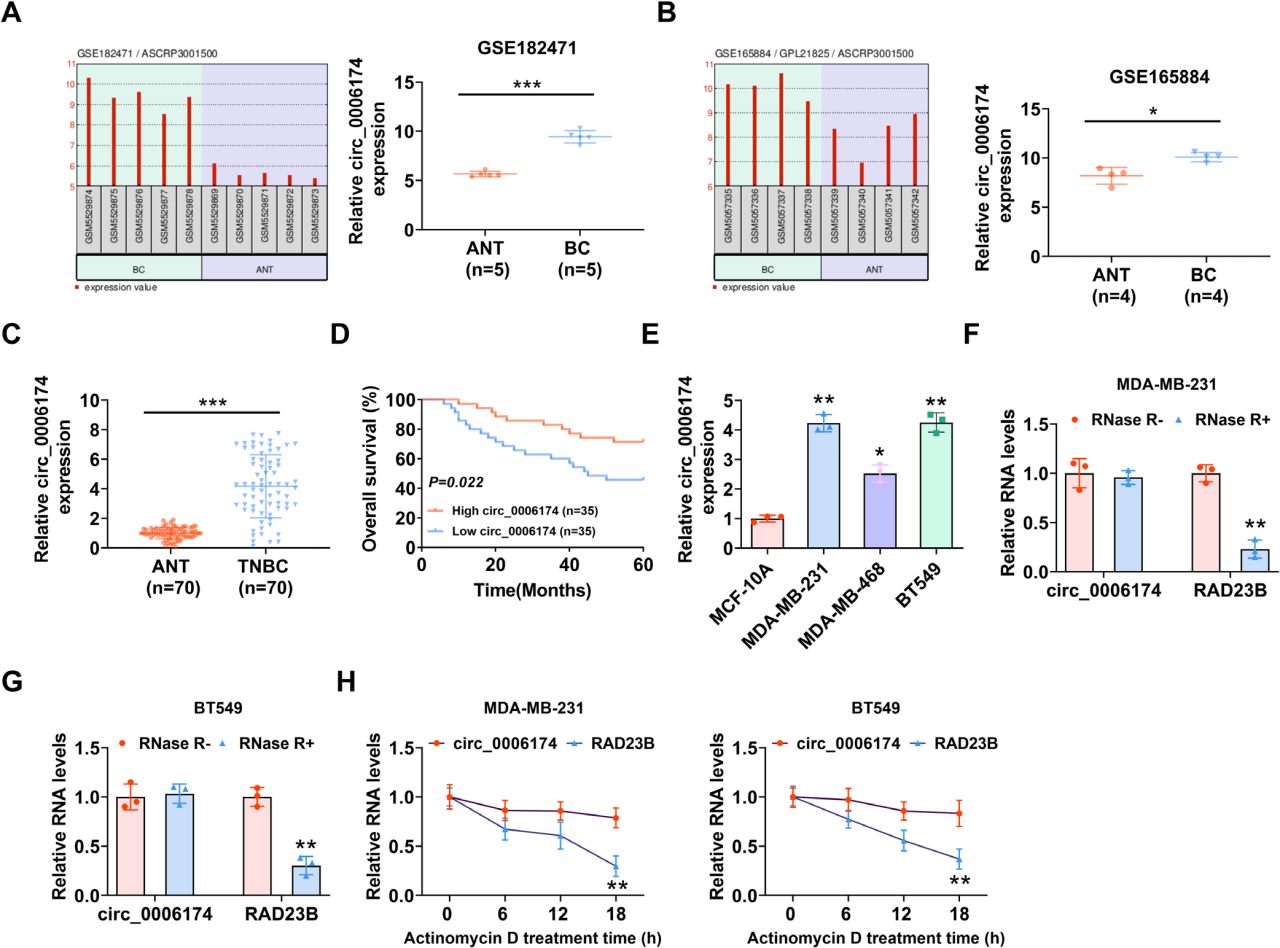
Upregulation of circ_0006174 indicated a poor prognosis in the TNBC patients. (A) Expression level of circ_0006174 in the tissues from five pairs of BC tissue samples and adjacent nontumor tissues based on the GSE182471 dataset was examined by the LIMMA package in R language. ***p < 0.001. (B) Expression level of circ_0006174 in the tissues from four pairs of BC tissue samples and adjacent nontumor tissues based on the GSE165884 dataset was determined by the LIMMA package in R language. *p < 0.05. (C) The expression level of *circ_0006174* was detected by RT‐qPCR in a pair of 70 TNBC tissues and adjacent nontumor tissues from TNBC patients. Data were expressed after being normalized with *GAPDH*. ***p < 0.001. (D) Seventy TNBC patients were divided into low circ_0006174 expression group (*n* = 35) and high circ_0006174 expression group (*n* = 35) based on the median circ_0006174 expression value in (C) as the cut‐off value, and OS of these two groups was evaluated by Kaplan–Meier survival curve. (E) The expression level of *circ_0006174* was examined by RT‐qPCR in MCF‐10A cells, as well as TNBC cell lines (MDA‐MB‐231, BT549 and MDA‐MB‐468 cells). Data were expressed after being normalized with *GAPDH*. *p < 0.05 and **p < 0.01 vs. MCF‐10A. The experiment was repeated three times, and data were represented as mean ± SD. Statistical differences were assessed with one‐way analysis of variance (ANOVA) followed by Post Hoc Bonferroni test. (F) The stability of circ_0006174 in comparison to linear RAD23B following RNase R treatment in MDA‐MB‐231 cells. **p < 0.01 vs. RNase R^−^. The experiment was repeated three times, and data were represented as mean ± SD. Statistical differences were determined with the Student's *t*‐test. (G) The stability of circ_0006174 in comparison to linear RAD23B following RNase R treatment in BT549 cells. **p < 0.01 vs. RNase R^−^. The experiment was repeated three times, and data were represented as mean ± SD. Statistical differences were determined with the Student's *t*‐test. (H) The stability of circ_0006174 in comparison to linear RAD23B following actinomycin D treatment in MDA‐MB‐231 and BT549 cells. **p < 0.01 vs. Actinomycin D treatment ^−^. The experiment was repeated three times, and data were represented as mean ± SD. Statistical differences were determined with the Student's *t*‐test.

To investigate the role of circ_0006174 in TNBC, its expression levels were initially measured in both TNBC tissues and cell lines. Analysis of datasets GSE182471 and GSE165884 revealed elevated circ_0006174 expression in the BC tissues (Figure 1A,B). To corroborate these findings, 70 paired samples of TNBC tissues and ANT were obtained from TNBC patients, demonstrating a significant increase in circ_0006174 expression in TNBC tissues (Figure 1C). Elevated circ_0006174 expression in TNBC patients was associated with shorter overall survival (OS) (Figure 1D), increased lymph node metastasis (Table 2), and higher TNM stage (Table 2) compared to patients with low circ_0006174 expression. In contrast, no statistically significant differences were observed in age, menopausal status, or tumor size between TNBC patients exhibiting high versus low expression levels of circ_0006174 (Table 2). Furthermore, elevated expression of circ_0006174 was detected in TNBC cell lines (Figure 1E). Given the higher expression levels of circ_0006174 in MDA‐MB‐231 and BT549 cells compared to MDA‐MB‐468 cells, MDA‐MB‐231 and BT549 cells were selected for further investigation. To verify the circular structure of circ_0006174, MDA‐MB‐231 and BT549 cells were treated with RNase R, an exonuclease that specifically degrades linear RNAs. Circ_0006174 exhibited significant resistance to RNase R digestion, whereas linear RAD23B mRNA was substantially degraded (Figure 1F,G). Transcriptional inhibition assays utilizing actinomycin D demonstrated that circ_0006174 had a half‐life exceeding 18 h, in contrast to a half‐life of less than 15 h for RAD23B mRNA (Figure 1H), highlighting its intrinsic stability. Furthermore, nuclear‐cytoplasmic fractionation verified that circ_0006174 was predominantly located in the cytoplasm of MDA‐MB‐231 and BT549 cells (Figure 1I). Collectively, these findings indicate that circ_0006174 is upregulated in TNBC and is associated with a poor prognosis for the patients with TNBC (Table 2).

**TABLE 2:** Correlation between circ_0006174 expression and the clinical pathological features of 70 triple negative breast cancer patients.

Characteristic	All cases	circ_0006174 expression		*p*
		Low (*n* = 35)	High (*n* = 35)	
Age (years)				0.629
< 50	30	16	14	
≥ 50	40	19	21	
Menopause				0.089
Yes	29	18	11	
No	41	17	24	
Tumor size (cm)				0.808
< 2	29	15	14	
≥ 2	41	20	21	
Lymph node metastasis				0.017*
No	34	22	12	
Yes	36	13	23	
TNM				0.016*
I + II	40	25	15	
III	30	10	20	

*Note*: A chi‐square test was used for comparing groups between low and high circ_0006174 expression.

* *p* < 0.05.

### 3.2 Knockdown of circ_0006174 Inhibited Proliferation and Invasion of TNBC Cells

**FIGURE 2:**
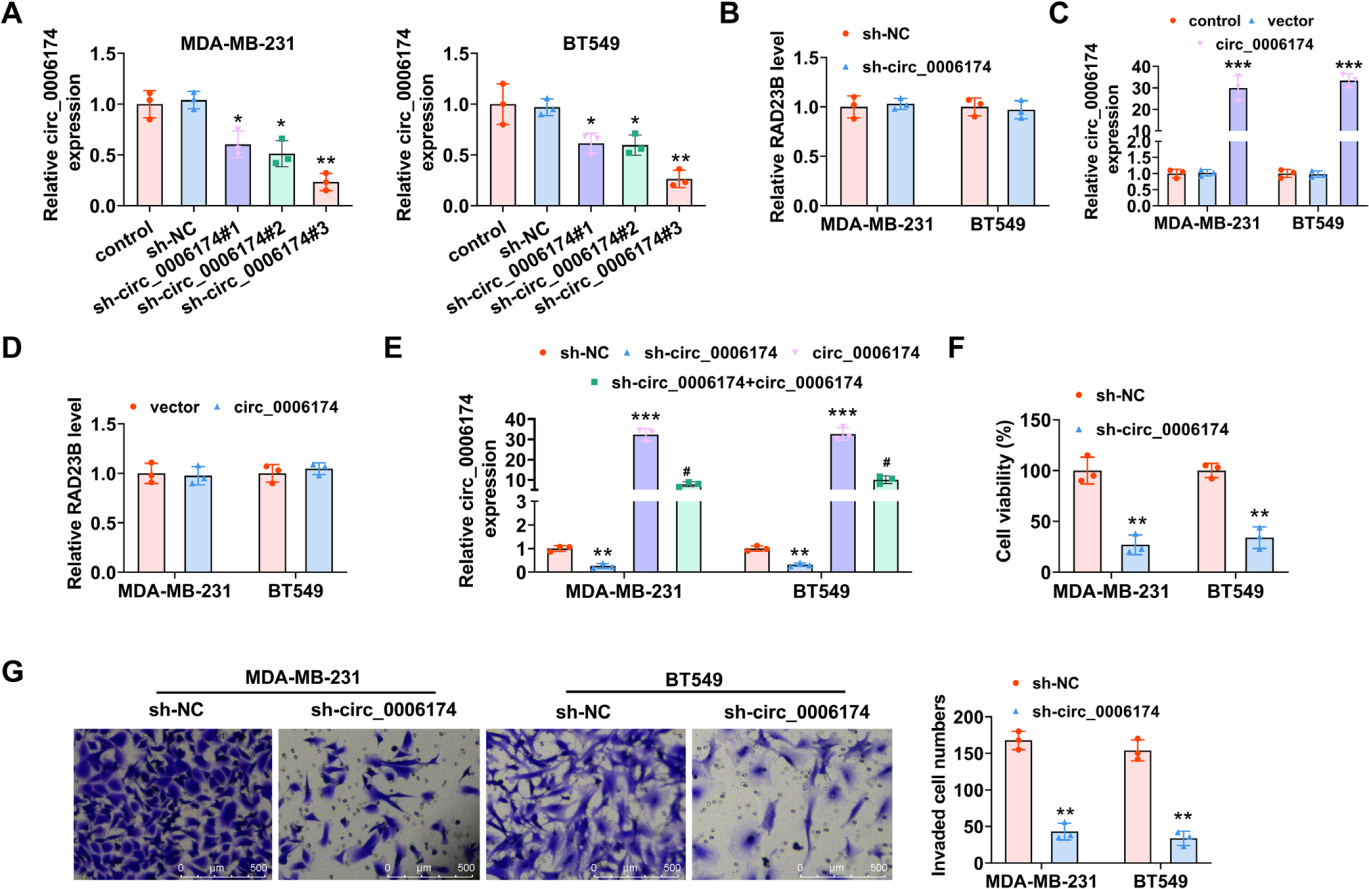
Interference of circ_0006174 suppressed the proliferation and invasion of TNBC cells. (A) Three shRNAs targeting circ_0006174 (sh‐circ_0006174#1, #2, and #3) were packaged into recombinant lentiviruses to knockdown the expression level of circ_0006174. The interfering efficiency of three sh‐circ_0006174 in MDA‐MB‐231 and BT549 cells was detected by RT‐qPCR. Data were expressed after being normalized with *GAPDH*. The experiment was repeated three times, and data were represented as mean ± SD. Statistical differences were assessed with one‐way analysis of variance (ANOVA) followed by Post Hoc Bonferroni test. (B) The interfering efficiency of sh‐circ_0006174 on the linear RAD23B was detected by RT‐qPCR. Data were expressed after being normalized with *GAPDH*. The experiment was repeated three times, and data were represented as mean ± SD. Statistical differences were determined with the Student's *t*‐test. (C) The overexpressing efficiency of the circ_0006174 overexpression plasmids was detected by RT‐qPCR. Data were expressed after being normalized with *GAPDH*. The experiment was repeated three times, and data were represented as mean ± SD. Statistical differences were assessed with one‐way analysis of variance (ANOVA) followed by Post Hoc Bonferroni test. (D) The overexpressing efficiency of the circ_0006174 overexpression plasmids on the linear RAD23B was detected by RT‐qPCR. Data were expressed after being normalized with *GAPDH*. The experiment was repeated three times, and data were represented as mean ± SD. Statistical differences were determined with the Student's *t*‐test. (E) The expression of circ_0006174 was detected by RT‐qPCR after MDA‐MB‐231 and BT549 cells were transfected with sh‐circ_0006174 or circ_0006174 overexpression plasmids. The experiment was repeated three times, and data were represented as mean ± SD. Statistical differences were assessed with one‐way analysis of variance (ANOVA) followed by Post Hoc Bonferroni test. (F) MDA‐MB‐231 and BT549 cells were transfected with sh‐circ_0006174, and the cell viability was examined by CCK‐8 assays. The experiment was repeated three times, and data were represented as mean ± SD. Statistical differences were determined with the Student's *t*‐test. (G) MDA‐MB‐231 and BT549 cells were transfected with sh‐circ_0006174, and the invasion ability was determined by transwell assays. Scale bar = 500 μm. *p < 0.05, **p < 0.01, and ***p < 0.001 vs. sh‐NC. #*p* < 0.05 vs. sh‐circ_0006174. The experiment was repeated three times, and data were represented as mean ± SD. Statistical differences were determined with the Student's *t*‐test.

To examine the role of circ_0006174 in the progression of TNBC, three shRNAs targeting circ_0006174 (designated as sh‐circ_0006174#1, #2, and #3) were employed to downregulate its expression in MDA‐MB‐231 and BT549 cell lines. All three shRNAs effectively reduced the levels of circ_0006174 in both cell lines compared to the sh‐NC (Figure 2A). Among these, sh‐circ_0006174#3 demonstrated the highest interference efficiency, surpassing that of sh‐circ_0006174#1 and #2. Consequently, sh‐circ_0006174#3 was selected for subsequent experiments and referred to as sh‐circ_0006174. No significant difference in circ_0006174 expression was observed between the sh‐NC group and the control group (Figure 2A). Furthermore, transfection with sh‐circ_0006174 did not significantly affect the expression levels of linear RAD23B mRNA (Figure 2B). Additionally, overexpression of circ_0006174 was performed in MDA‐MB‐231 and BT549 cells (Figure 2C). Similarly, this overexpression did not significantly alter the expression of linear RAD23B mRNA (Figure 2D). Notably, transfection with sh‐circ_0006174 led to a decrease in circ_0006174 expression, which was subsequently restored upon overexpression of circ_0006174 (Figure 2E). The elevated expression of circ_0006174 was effectively mitigated through the transfection of sh‐circ_0006174 (Figure 2E). This transfection resulted in a reduction in cell viability and the number of invaded cells in both MDA‐MB‐231 and BT549 cells (Figure 2F,G). Overall, the interference with circ_0006174 inhibited the proliferation and invasion of TNBC cells.

### 3.3 Silencing of circ_0006174 Repressed the Immune Escape of TNBC

**FIGURE 3:**
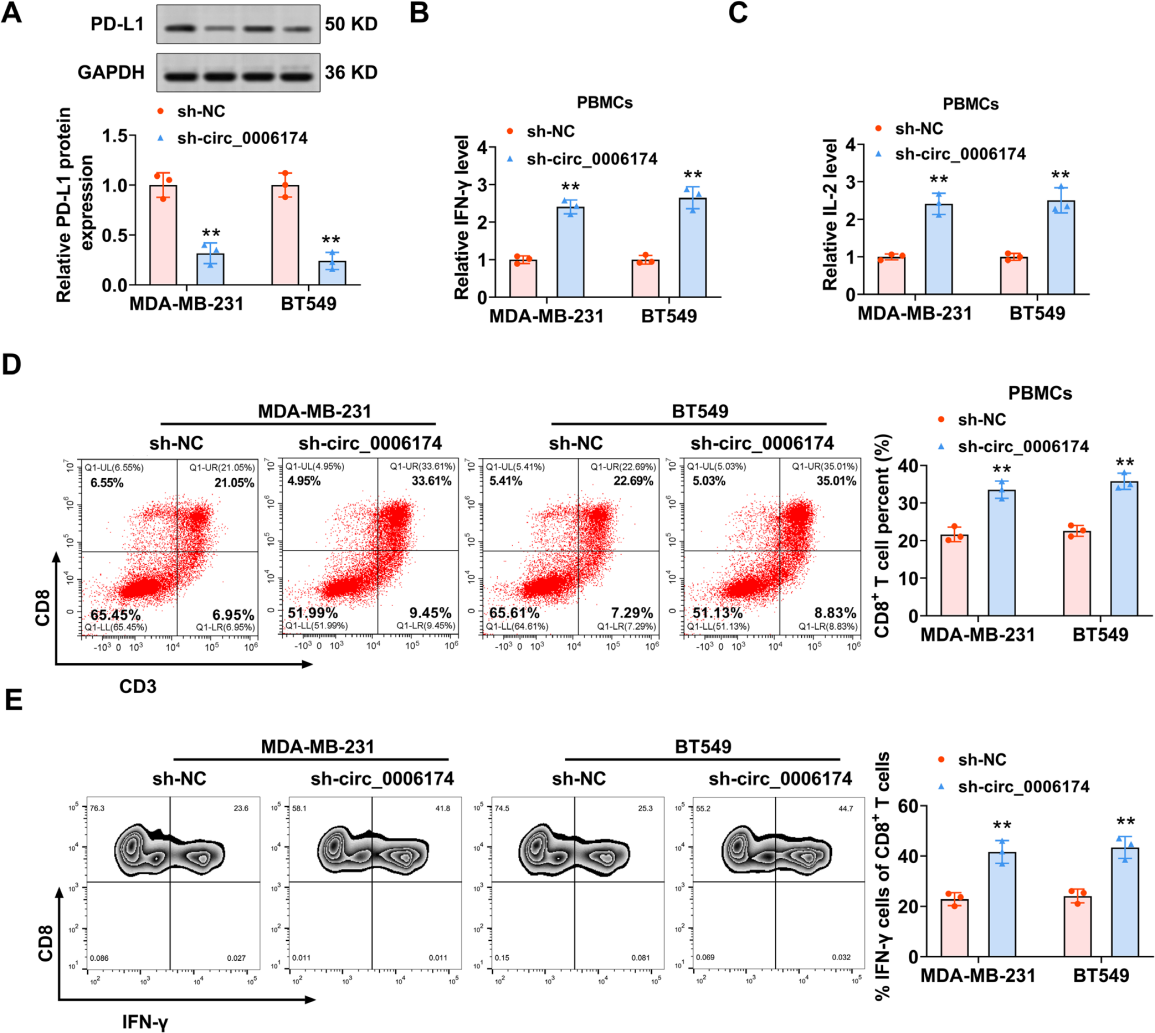
Interference of circ_0006174 suppressed the immune escape of TNBC cells. MDA‐MB‐231 and BT549 cells were transfected with sh‐circ_0006174. CD8^+^ T cells were activated with IL‐2 and Human T‐Activator CD3/CD28 Dynabeads for 2 weeks. Then, transfected MDA‐MB‐231 and BT549 cells were grown with CD8^+^ T cells for 24 h. The media of co‐culture cells were collected for the following measurements. (A) The expression of PD‐L1 in transfected MDA‐MB‐231 and BT549 cells was examined by western blot. Data were expressed after being normalized with GAPDH. (B) Measurement of the concentrations of IFN‐γ in the media of co‐culture cells by ELISA. (C) Measurement of the concentrations of IL‐2 in the media of co‐culture cells by ELISA. (D) The percent of CD8^+^ T cells was detected by flow cytometry. (E) The percent of IFN‐γ cells in CD8^+^ T cells was detected by flow cytometry. **p < 0.01 vs. sh‐NC. The experiment was repeated three times, and data were represented as mean ± SD. Statistical differences were determined with the Student's *t*‐test.

Furthermore, the level of PD‐L1 was reduced in MDA‐MB‐231 and BT549 cells following transfection with sh‐circ_0006174 (Figure 3A). To further substantiate its role, transfected MDA‐MB‐231 and BT549 cells were co‐cultured with activated CD8^+^ T cells. The knockdown of circ_0006174 led to an increase in the concentrations of IFN‐γ and IL‐2 in the co‐culture media (Figure 3B,C), as well as an increase in the percentage of CD8^+^ T cells (Figure 3D). Additionally, the inhibition of circ_0006174 elevated the percentage of IFN‐γ‐producing cells among the CD8^+^ T cells (Figure 3E). In summary, the knockdown of circ_0006174 suppressed the immune escape of TNBC.

### 3.4 Circ_0006174 Positively Regulated the Protein Expression of PD‐L1 by Targeting miR‐3139

**FIGURE 4:**
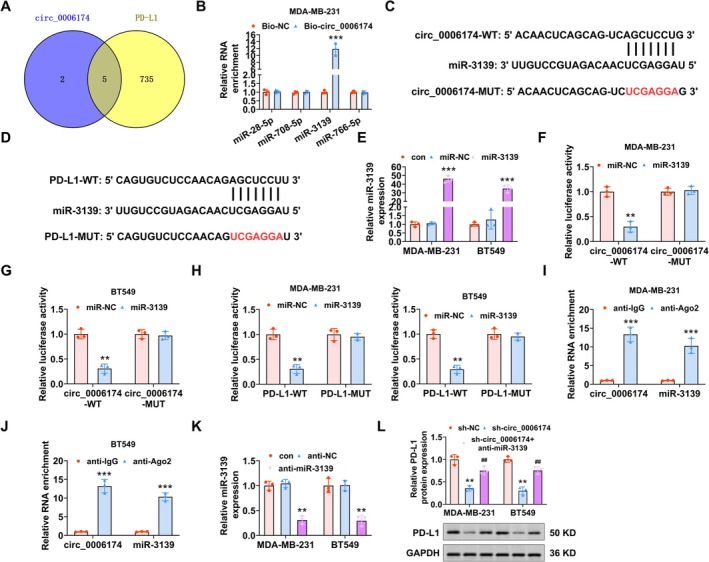
Circ_0006174 positively regulated the protein expression of PD‐L1 by targeting miR‐3139. (A) The potential miRNAs targeted by circ_0006174 were predicted by the Starbase online platform, and the potential miRNAs targeted by PD‐L1 were predicted by the Targetscan database platform. After the miRNAs targeted by circ_0006174 intersected with the miRNAs targeted by PD‐L1, there were five miRNAs, including hsa‐miR‐138‐5p, hsa‐miR‐28‐5p, hsa‐miR‐708‐5p, hsa‐miR‐3139, and hsa‐miR‐766‐5p. (B) RNA pull‐down results exhibited that the biotinylated circ_0006174 probes enriched for miR‐3139. ***p < 0.001 vs. Bio‐NC. The experiment was repeated three times, and data were represented as mean ± SD. Statistical differences were determined with the Student's *t*‐test. (C) The binding sites between circ_0006174 and miR‐3139 were predicted by the Starbase online platform, and the wild‐type and mutant sequences of circ_0006174 were also designed and exhibited. (D) The binding sites between miR‐3139 and PD‐L1 were predicted by the Targetscan online platform, and the wild‐type and mutant sequences of PD‐L1 were also designed and exhibited. (E) miR‐3139 mimics were transfected into MDA‐MB‐231 and BT549 cells by Lipofectamine 3000. The overexpression efficiency of miR‐3139 was detected by qRT‐PCR. The data were normalized with *U6*. ***p < 0.001 vs. miR‐NC. The experiment was repeated three times, and data were represented as mean ± SD. Statistical differences were assessed with one‐way analysis of variance (ANOVA) followed by Post Hoc Bonferroni test. (F) The luciferase activities were determined in MDA‐MB‐231 cells by the dual luciferase reporter assay system to confirm the direct relationship between circ_0006174 and miR‐3139. **p < 0.01 vs. miR‐NC. The experiment was repeated three times, and data were represented as mean ± SD. Statistical differences were determined with the Student's *t*‐test. (G) The luciferase activities were determined in BT549 cells by the dual luciferase reporter assay system to confirm the direct relationship between circ_0006174 and miR‐3139. **p < 0.01 vs. miR‐NC. The experiment was repeated three times, and data were represented as mean ± SD. Statistical differences were determined with the Student's *t*‐test. (H) The luciferase activities were determined in MDA‐MB‐231 and BT549 cells by the dual luciferase reporter assay system to confirm the direct relationship between miR‐3139 and PD‐L1. **p < 0.01 vs. miR‐NC. The experiment was repeated three times, and data were represented as mean ± SD. Statistical differences were determined with the Student's *t*‐test. (I) RIP results showed that the Ago2 group enriched more circ_0006174 and miR‐3139 compared to the IgG group in MDA‐MB‐231 cells. ***p < 0.001 vs. anti‐IgG. The experiment was repeated three times, and data were represented as mean ± SD. Statistical differences were determined with the Student's *t*‐test. (J) RIP results showed that the Ago2 group enriched more circ_0006174 and miR‐3139 compared to the IgG group in BT549 cells. ***p < 0.001 vs. anti‐IgG. The experiment was repeated three times, and data were represented as mean ± SD. Statistical differences were determined with the Student's *t*‐test. (K) miR‐3139 inhibitors were transfected into MDA‐MB‐231 and BT549 cells by Lipofectamine 3000. The knockdown efficiency of miR‐3139 was detected by qRT‐PCR. The data were normalized with *U6*. **p < 0.01 vs. anti‐NC. The experiment was repeated three times, and data were represented as mean ± SD. Statistical differences were assessed with one‐way analysis of variance (ANOVA) followed by Post Hoc Bonferroni test. (L) The relative protein expression of PD‐L1 was detected by western blot after MDA‐MB‐231 and BT549 cells were transfected with sh‐circ_0006174 or/and miR‐3139 inhibitors. Data were normalized with GAPDH. **p < 0.01 vs. sh‐NC; ##*p* < 0.01 vs. sh‐circ_0006174. The experiment was repeated three times, and data were represented as mean ± SD. Statistical differences were assessed with one‐way analysis of variance (ANOVA) followed by Post Hoc Bonferroni test.

circRNAs typically function as competing endogenous RNAs (ceRNAs) to regulate gene expression by acting as molecular sponges for miRNA [11]. The potential miRNAs were identified using the Starbase online platform. In investigating the role of circ_0006174 in the immune escape of BC, the miRNAs potentially targeted by PD‐L1 were identified using the TargetScan database. Upon intersecting results, five miRNAs were identified: miR‐138‐5p, miR‐28‐5p, miR‐708‐5p, miR‐3139, and miR‐766‐5p (Figure 4A). Among these, the association between circ_0006174 and miR‐138‐5p has been previously documented. Consequently, the interactions between circ_0006174 and the remaining four miRNAs were investigated through RNA pull‐down assays. The biotinylated circ_0006174 probes specifically enriched miR‐3139 compared to the NC probes (Figure 4B). The complementary base pairings between circ_0006174 and miR‐3139, as well as between miR‐3139 and PD‐L1, were illustrated in Figure 4C,D, which also displayed the mutant sequences of circ_0006174 and PD‐L1. To validate the interaction between circ_0006174 and miR‐3139, miR‐3139 expression was upregulated in MDA‐MB‐231 and BT549 cells through transfection with miR‐3139 mimics (Figure 4E). The co‐transfection of miR‐3139 mimics with circ_0006174‐WT resulted in a reduction of relative luciferase activity, whereas co‐transfection with circ_0006174‐MUT did not affect relative luciferase activity in either cell line (Figure 4F,G). The results demonstrated a direct binding between circ_0006174 and miR‐3139. Similar binding interactions were observed between miR‐3139 and PD‐L1 (Figure 4H). Furthermore, the Ago2 group demonstrated a higher enrichment of circ_0006174 and miR‐3139 compared to the IgG group in both MDA‐MB‐231 and BT549 cells (Figure 3I,J). The administration of anti‐miR‐3139 led to a decrease in miR‐3139 levels (Figure 3K). Silencing circ_0006174 reduced PD‐L1 levels in both cell lines, an effect that was reversed by the application of anti‐miR‐3139 (Figure 3L). Altogether, these findings suggest that circ_0006174 positively regulated the PD‐L1 expression by targeting miR‐3139.

### 3.5 Circ_0006174 Regulated the PD‐L1 Expression to Promote the Cell Growth, Invasion and Immune Evasion via miR‐3139 in TNBC

**FIGURE 5:**
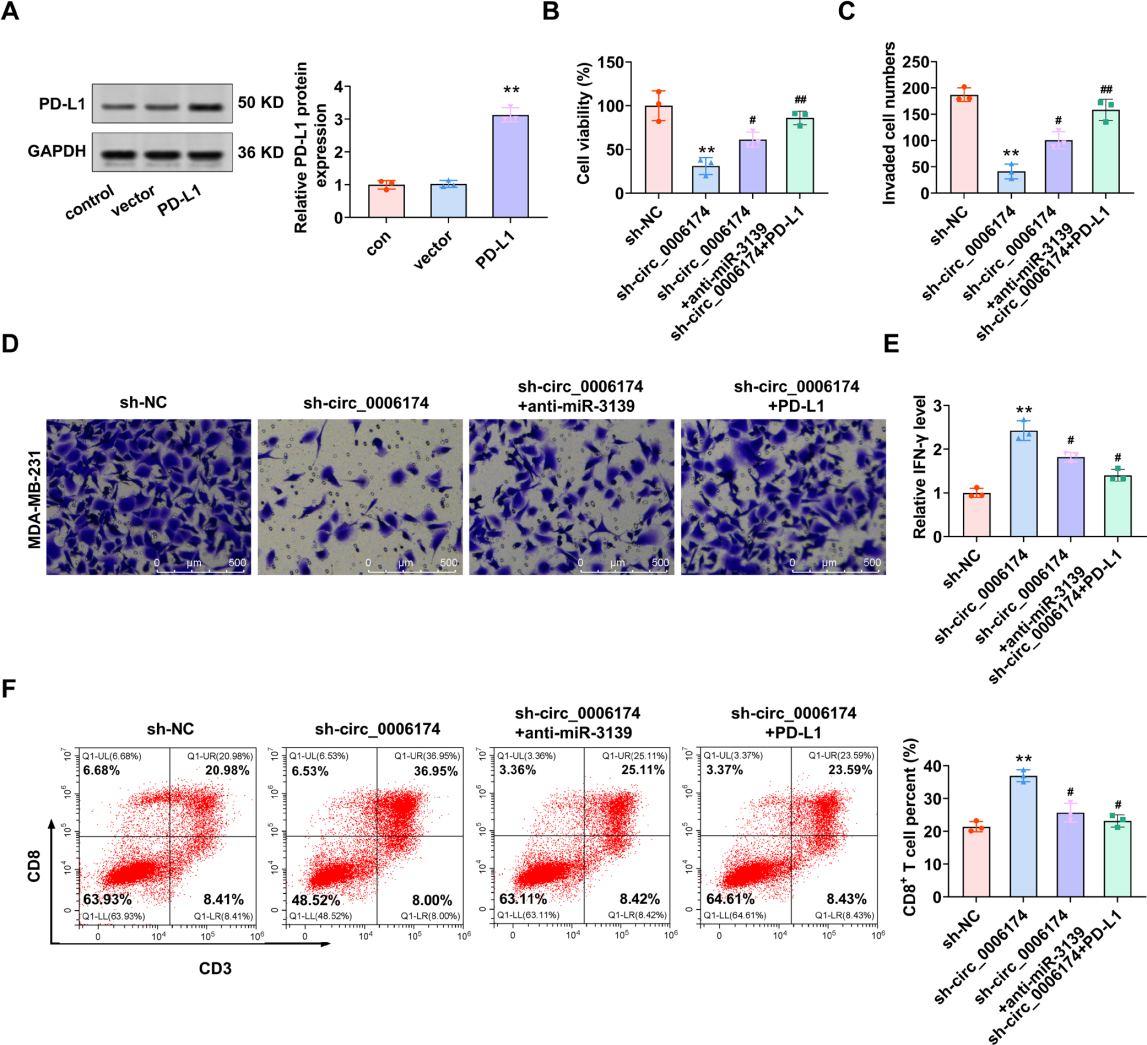
Knockdown of circ_0006174 repressed cell viability, invasion, and immune escape by the miR‐3139/PD‐L1 axis in TNBC. (A) The PD‐L1 sequences were inserted into pcDNA vector plasmids and then transfected into MDA‐MB‐231 cells by Lipofectamine 3000. The overexpression efficiency of PD‐L1 was detected by western blot. Data were normalized with GAPDH. **p < 0.01 vs. vector. (B) MDA‐MB‐231 cells were transfected with sh‐circ_0006174, sh‐circ_0006174 and miR‐3139 inhibitors, or sh‐circ_0006174 and PD‐L1 overexpression plasmids. MDA‐MB‐231 cell viability was determined by CCK‐8 assays. **p < 0.01 vs. sh‐NC; #*p* < 0.05 and ##*p* < 0.01 vs. sh‐circ_0006174. (C and D) The invaded cell numbers of MDA‐MB‐231 cells were calculated by transwell experiments. Scale bar = 500 μm. **p < 0.01 vs. sh‐NC; #*p* < 0.05 and ##*p* < 0.01 vs. sh‐circ_0006174. (E) MDA‐MB‐231 cells were transfected with sh‐circ_0006174, sh‐circ_0006174 and miR‐3139 inhibitors, or sh‐circ_0006174 and PD‐L1 overexpression plasmids. Transfected MDA‐MB‐231 cells were grown with activated CD8^+^ T cells for 24 h. The concentrations of IFN‐γ in the media of co‐culture cells were detected by ELISA. **p < 0.01 vs. sh‐NC; #*p* < 0.05 vs. sh‐circ_0006174. (F) The percent of CD8^+^ T cells was detected by flow cytometry. **p < 0.01 vs. sh‐NC; #*p* < 0.05 vs. sh‐circ_0006174. The experiment was repeated three times, and data were represented as mean ± SD. Statistical differences were assessed with one‐way analysis of variance (ANOVA) followed by the Post Hoc Bonferroni test.

To further investigate the role of circ_0006174 in the malignant progression of TNBC via the miR‐3139/PD‐L1 axis, PD‐L1 expression was upregulated in MDA‐MB‐231 cells (Figure 5A). Transfection with sh‐circ_0006174 in MDA‐MB‐231 cells led to a decrease in cell viability and invasion, effects that were reversed by miR‐3139 knockdown or PD‐L1 overexpression (Figure 5B–D). Furthermore, the silencing of miR‐3139 or the overexpression of PD‐L1 counteracted the elevation in IFN‐γ levels and the proportion of CD8^+^ T cells following the co‐treatment of PBMCs with MDA‐MB‐231 cells transfected with sh‐circ_0006174 (Figure 5E,F). Overall, circ_0006174 regulated PD‐L1 expression to promote TNBC cell growth, invasion, and immune evasion via miR‐3139.

### 3.6 circ_0006174 Was Modulated by m6A Methylation

**FIGURE 6:**
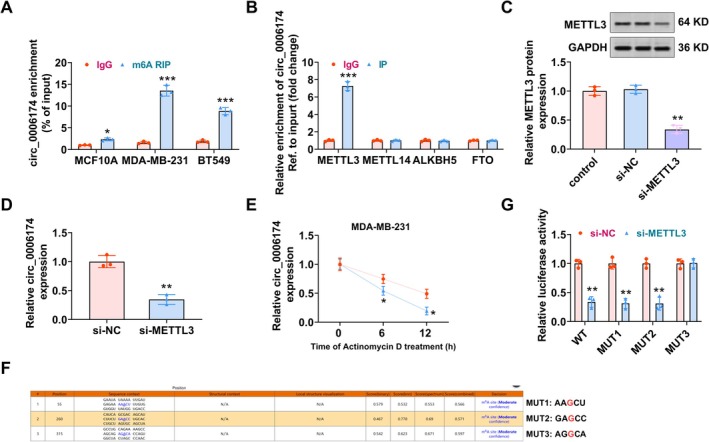
circ_0006174 was modulated by m6A methylation. (A) m6A RIP‐qPCR analysis of circ_0006174 in MCF10A, MDA‐MB‐231, and BT549 cells. *p < 0.05 and ***p < 0.001 vs. IgG. (B) RIP assay was used to detect which m6A enzymes were involved in the regulation of circ_0006174 in MDA‐MB‐231 cells. ***p < 0.001 vs. IgG. (C) The knockdown efficiency of METTL3 in MDA‐MB‐231 cells was detected by western blot. Data were normalized with GAPDH. **p < 0.01 vs. si‐NC. (D) qRT‐PCR to detect circ_0006174 expression in MDA‐MB‐231 cells with silencing of METTL3. Data were expressed after being normalized with *GAPDH*. **p < 0.01 vs. si‐NC. (E) The stability of circ_0006174 was assessed after MDA‐MB‐231 cells were downregulated with METTL3 following actinomycin D treatment at 0, 3, 6, and 9 h. *p < 0.05 vs. si‐NC. (F) The m6A sites of circ_0006174 were predicted through SRAMP (http://www.cuilab.cn/sramp), and the mutated m6A sites (M1–M3) circ_0006174 sequences were exhibited. (G) The constructed luciferase reporter plasmids by inserting partial wildtype circ_0006174 sequences or mutated m6Asites (M1‐M3) circ_0006174 sequences. The luciferase reporters with wildtype or mutant plasmids were transfected into MDA‐MB‐231 cells (with the downregulated METTL3), followed by the measurement of luciferase activity. **p < 0.01 vs. si‐NC. The experiment was repeated three times, and data were represented as mean ± SD. Statistical differences were determined with the Student's *t*‐test.

Notably, N6‐methyladenosine (m6A) has been identified as the most prevalent modification in noncoding RNAs, playing a crucial role in circRNA biogenesis [21]. m6A‐specific immunoprecipitation assays revealed elevated m6A levels on circ_0006174 across MCF10A, MDA‐MB‐231, and BT549 cells, with TNBC cells exhibiting higher m6A levels compared to MCF10A cells (Figure 6A). This suggested that m6A modification might contribute to the upregulation of circ_0006174. To further elucidate the molecular mechanisms underlying the m6A modification of circ_0006174, a RIP assay was conducted to identify the m6A enzymes involved in the regulation of circ_0006174 in MDA‐MB‐231 cells. The findings indicated that METTL3 was capable of enriching the RNA fragments of circ_0006174, whereas METTL14, ALKBH5, and FTO did not demonstrate a significant binding affinity for these RNA fragments (Figure 6B). Consequently, METTL3 is likely the primary m6A modifying enzyme for circ_0006174. Subsequent investigations revealed a marked reduction in the expression of circ_0006174 in METTL3‐downregulated MDA‐MB‐231 cells (Figure 6C,D). Actinomycin D assays further indicated that the stability of circ_0006174 was compromised following METTL3 knockdown (Figure 6E). To pinpoint the specific m6A methylation sites on circ_0006174, SRAMP software (http://www.cuilab.cn/sramp/) was used to predict its high‐confidence m6A sites. Three m6A sites on circ_0006174 were predicted using the SRAMP platform (Figure 6F). Subsequently, luciferase reporter plasmids were constructed by incorporating partial sequences of circ_0006174 containing either wild‐type or mutant m6A sites (MUT1: mutant site 55; MUT2: mutant site 260; MUT3: mutant site 315) (Figure 6F). Upon downregulation of METTL3, a reduction in luciferase activity was observed in MDA‐MB‐231 cells transfected with both wild‐type and mutant plasmids (MUT1 and MUT2) (Figure 6G). In contrast, the luciferase activity of circ_0006174 containing the MUT3 mutation remained unchanged (Figure 6G). This suggested that the regulation of circ_0006174 by METTL3 was contingent upon m6A methylation, with the primary m6A methylation site identified at position 315.

### 3.7 Knockdown circ_0006174 Suppressed TNBC Cell Growth and Immune Escape In Vivo

**FIGURE 7:**
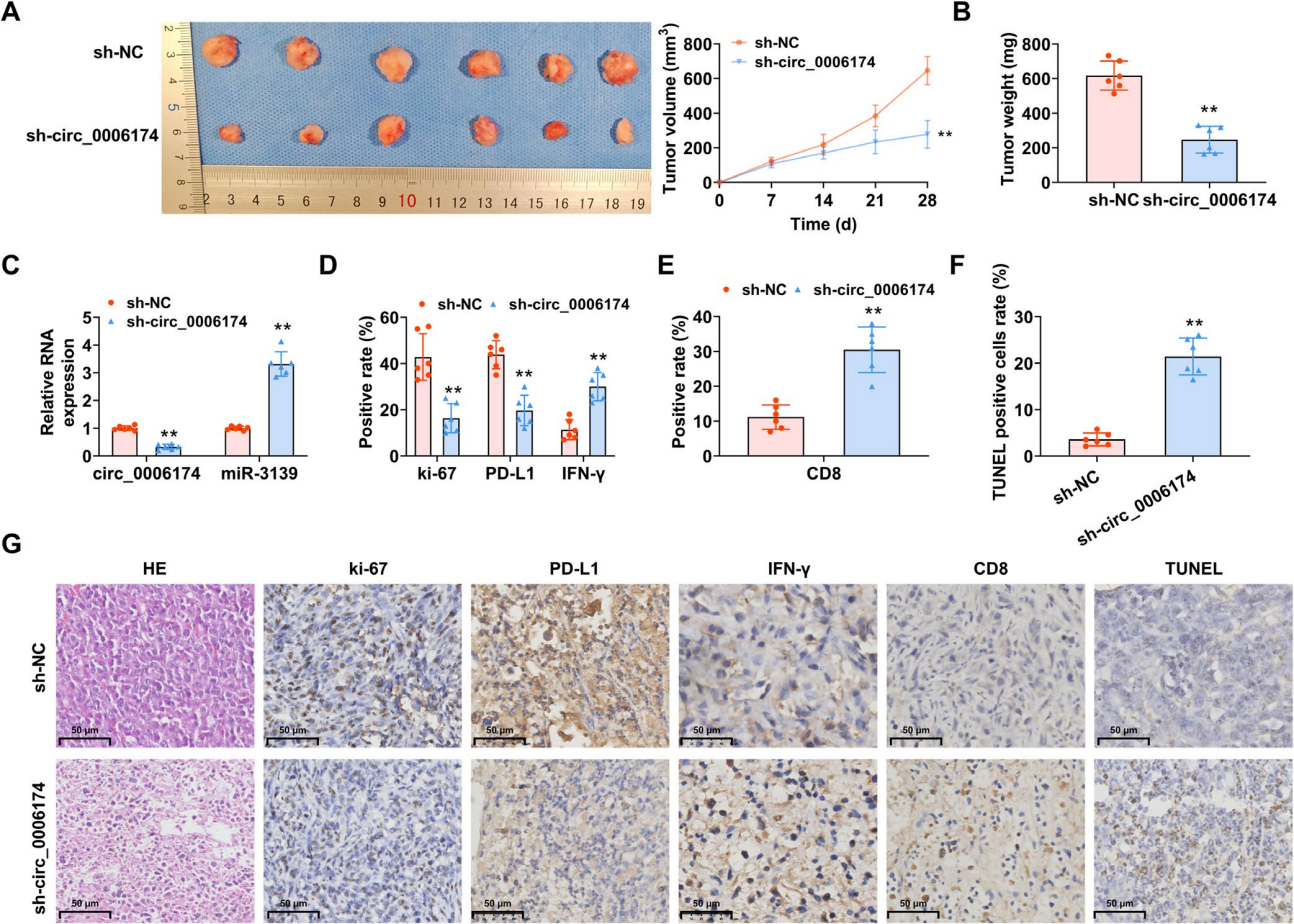
Downregulation of circ_0006174 inhibited TNBC malignant progression involving the miR‐3139/PD‐L1 axis in vivo. 7‐Hu‐PBL‐NSG mice were subcutaneously inoculated with a total of 2 × 10^6^ MDA‐MB‐231 cells with lentivirus packaged sh‐NC or sh‐circ_0006174 in the left flank region. Tumor volume was monitored 7 days for four consecutive weeks and quantified by the following formula: Volume = 0.5 × length×width^2^. After 5 weeks, mice were sacrificed, and the tumor samples were collected and weighed. (A) Representative images of neoplasms from mice (Left). Monitor of tumor volume every week for four successive weeks (Right). (B) Measurement of tumor weight after 5 weeks. (C) The expression level of *circ_0006174* and *miR‐3139* was detected by RT‐qPCR in tissues from mice. Data were expressed after being normalized with *GAPDH* or *U6*. (D and E) The expression levels of ki‐67, PD‐L1, IFN‐γ, and CD8 in tumor tissues were detected by immunohistochemistry and exhibited as histograms. (F) The TUNEL positive cell rate was examined by TUNEL staining and exhibited as a histogram. (G) Pathological changes were examined by HE staining, the expression levels of ki‐67, PD‐L1, IFN‐γ, and CD8 in tumor tissues were detected by immunohistochemistry, and apoptosis was assessed by TUNEL staining. Scale bar = 50 μm. **p < 0.01 vs. sh‐NC. The experiment was repeated three times, and data were represented as mean ± SD. Statistical differences were determined with the Student's *t*‐test.

Furthermore, the role of circ_0006174 was investigated in vivo using lentivirus‐packaged sh‐circ_0006174. Mice treated with sh‐circ_0006174 exhibited reduced tumor size and weight compared to those treated with sh‐NC (Figure 7A,B). The knockdown of circ_0006174 resulted in increased expression of miR‐3139 (Figure 7C). Immunohistochemical analysis revealed that inhibition of circ_0006174 led to decreased levels of ki‐67 and PD‐L1 while increasing the levels of IFN‐γ and CD8 (Figure 7D,E,G). Additionally, suppression of circ_0006174 enhanced the rate of TUNEL‐positive cells in vivo (Figure 7F,G). Pathological examination further indicated that mice treated with sh‐NC displayed loosely arranged cells with an irregular pattern, which was significantly improved following the knockdown of circ_0006174 (Figure 7G). The silencing of circ_0006174 was found to inhibit BC cell growth and immune evasion involved in the miR‐3139/PD‐L1 axis in mice. Collectively, circ_0006174, which was regulated by METTL3‐mediated m6A methylation, promoted proliferation, invasion, and immune escape in TNBC via the miR‐3139/PD‐L1 axis.

## 4 Discussion

This study identified a correlation between circ_0006174 overexpression and poor prognosis in TNBC patients. Genetic silencing of circ_0006174 resulted in the suppression of proliferation, invasion, and PD‐L1‐mediated immune modulation in TNBC. At the molecular level, circ_0006174 acted as a sponge for miR‐3139, leading to the upregulation of PD‐L1. The regulation of circ_0006174 by METTL3 was dependent on m6A methylation at the site of 315. In vivo silencing of circ_0006174 led to a reduction in tumor burden and decreased expression of ki‐67 and PD‐L1, while increasing the levels of miR‐3139 and IFN‐γ. Overall, circ_0006174, regulated by METTL3‐mediated m6A methylation, was shown to promote TNBC cell growth, invasion, and immune evasion through the miR‐3139/PD‐L1axis.

Given that certain circRNAs are dysregulated in TNBC, they present potential targets for diagnosis and treatment [8]. Circ_0006174 has been demonstrated to be abundantly present in colorectal cancer tissues and cells [14, 15, 16, 17, 18]. Furthermore, it has been established that circ_0006174 is negatively associated with the overall survival of colorectal cancer patients and positively correlated with TNM stage and lymph node metastases [18]. In alignment with observations, our study found that circ_0006174 expression was elevated in TNBC cell lines and tissues, and it was associated with a higher TNM stage, reduced OS, and increased lymph node metastases in TNBC patients. Consequently, these findings suggest that overexpression of circ_0006174 in TNBC is indicative of a poor prognosis for patients, highlighting its potential as a therapeutic target.

The dysregulation of circ_0006174 in TNBC implies its relevance to the progression of the disease. Numerous studies have demonstrated that circRNAs play a significant role in the progression of BC [22, 23]. In this study, silencing circ_0006174 resulted in decreased cell viability in both BT549 and MDA‐MB‐231 cells. Moreover, the knockdown of circ_0006174 resulted in a reduction in tumor weight and size, as well as a decrease in the ki‐67 level in tumor tissues derived from mice implanted with MDA‐MB‐231 cell xenografts. Ki‐67 is widely recognized as a standard marker for assessing cell proliferation rates. These results indicated that silencing circ_0006174 effectively inhibits the proliferation of triple‐negative breast cancer (TNBC) cells in both in vitro and in vivo models. Additionally, the downregulation of circ_0006174 led to a reduction in the number of invading cells in TNBC cell lines, suggesting that circ_0006174 silencing impeded TNBC cell invasion. A fundamental and notable characteristic of all cancer types, including TNBC, is their ability to invade and proliferate. It has been demonstrated that circRNAs play a role in various aspects of TNBC, including invasion and proliferation [24]. Furthermore, the knockdown of circ_0006174 has been shown to suppress the proliferation and invasion of colorectal cancer cells [14, 15, 17, 18]. Overall, these findings underscored that the downregulation of circ_0006174 inhibited TNBC cell invasion and proliferation.

Additionally, immune evasion is a critical factor in the progression of malignancies. The study has demonstrated that chemicals capable of preventing immune escape are significant and promising candidates for the treatment of TNBC [25]. In the present study, knockdown of circ_0006174 resulted in a reduction of, while simultaneously enhancing the cytotoxicity of CD8+ T cells and increasing the levels of IFN‐γ and IL‐2 in two TNBC cell lines. Moreover, the downregulation of circ_0006174 led to decreased PD‐L1 expression and elevated IFN‐γ expression in mice. The tumor microenvironment (TME), which consists of a variety of immune populations, stromal components, malignant cells, and signaling molecules, plays a crucial role in regulating carcinogenesis by affecting tumor progression, metastatic potential, and responses to immunomodulatory therapies [26]. Within this intricate ecosystem, the eradication of tumors primarily relies on adaptive immunity mediated by effector lymphocytes, particularly the CD4+ and CD8+ T cell subsets. Activated CD4+ T lymphocytes differentiate into helper subtypes that secrete IL‐2, thereby enhancing the activity of potentiating cytotoxic CD8+ T lymphocytes (CTLs), which are responsible for the targeted destruction of tumor cells [27]. Progressive tumorigenesis leads to functional impairment in CTLs through the induction of immunosuppressive mechanisms and tolerance within the TME. Therapeutic interventions targeting these exhaustion pathways hold the potential to restore CD8+ T cell functionality, thereby facilitating the sustained elimination of antigen‐presenting malignancies. The immune checkpoint molecule PD‐L1 is pivotal in tumor immune evasion strategies. Recent studies indicate that malignant cells and their microenvironment can suppress T‐cell activation pathways by enhancing PD‐1/PD‐L1 axis signaling through increased PD‐L1 expression, and ultimately leading to apoptosis in effector T cells [28]. CD4+ T lymphocytes demonstrate functional diversity in anti‐tumor immunity via distinct cytokine secretion profiles [29]. Th1 cells predominantly produce IFN‐γ, whereas Th2, Th3, and Tr1 subsets secrete IL‐4, IL‐5, IL‐10, and TGF‐β, respectively. This functional dichotomy results in Th1‐mediated cellular immunity as opposed to the promotion of humoral responses by other subsets, rendering the Th1/Th2/Th3/Tr1 cytokine balance critical in determining immune response outcomes. Therapeutically beneficial anti‐tumor responses are associated with Th1 dominance, whereas a predominance of Th2/Th3/Tr1 is linked to tumor progression. The immunological shift from a Th1 to alternative cytokine profiles, referred to as “Th1‐Th2 axis polarization,” serves as a critical biomarker indicating compromised cellular defense mechanisms against tumors during carcinogenesis [30]. Overall, these findings demonstrate that the knockdown of circ_0006174 reduced the ability of TNBC cells to evade the immune system.

A prominent characteristic of circRNAs is their capacity to modulate gene expression by functioning as molecular sponges for miRNA through the ceRNA mechanism [11]. Emerging evidence suggests that circRNAs regulate the development and progression of BC via the ceRNA mechanism, according to accumulating data. For instance, Xu et al. [31] have demonstrated that circPOLA2 acts as a sponge for miR‐1224‐5p to regulate HMGA2 expression, thereby facilitating the proliferation and invasion of BC. Jin and colleagues [32] have reported that circFKBP8 promotes BC cell growth and invasion through the miR‐432‐5p/E2F7 axis. Additionally, circFGFR4 enhances the immune escape of TNBC cells via the miR‐185‐5p/CXCR4 axis [20]. In this study, miR‐3139 was identified as a target of circ_0006174, with evidence of direct interaction demonstrated through pull‐down, luciferase reporter, and RIP assays. MiR‐3139 expression is reduced in TNBC tissues and cells [33]. Additionally, miR‐3139 has been shown to be sequestered by non‐coding RNAs, which regulate the expression of target genes and thereby influence the progression and development of BC. For instance, the long noncoding RNA LINC00514 acts as a sponge for miR‐3139, modulating CCDC71L expression to promote TNBC cell growth, mobility, and invasion [33]. Further analysis in this study revealed that miR‐3139 can directly bind to PD‐L1. PD‐L1 is overexpressed in BC and is significantly associated with malignant progression and poor prognosis in BC patients [34, 35]. Moreover, this study found that the knockdown of circ_0006174 led to reduced PD‐L1 expression in TNBC cells, an effect that was reversed by treatment with anti‐miR‐3139. Furthermore, the inhibitory effects of sh‐circ_0006174 on TNBC cell proliferation, invasion, and immune escape were negated by either miR‐3139 knockdown or PD‐L1 overexpression. PD‐L1 is predominantly recognized as an immune suppressive molecule, lacking a direct role in enhancing tumor cell proliferation or invasion. In this study, we elucidated that PD‐L1 served as a downstream target of circ_0006174, with circ_0006174 regulating PD‐L1 expression to facilitate immune evasion in TNBC cells, thereby exerting an anti‐tumor effect. Previous studies have documented that PD‐L1 expression can be regulated by various circRNAs. For instance, circ_0067842 promotes tumor metastasis and immune escape in breast cancer through the HuR/CMTM6/PD‐L1 axis [36]. Similarly, circGSK3β influences PD‐L1 transcription via the miR‐338‐3p/PRMT5/H3K4me3 pathway, thereby enhancing immune evasion and tumor progression in BC [13]. Furthermore, it is plausible that additional oncogenic targets may collaborate with PD‐L1 to drive tumor progression, necessitating further research in this area. Altogether, these findings indicated that circ_0006174 regulated PD‐L1 expression, thereby promoting TNBC cell growth, invasion, and immune evasion through the miR‐3139 pathway.

The m6A modification is facilitated by m6A writers, including METTL3, METTL14, KIAA1429, WTAP, RBM15, and ZC3H13; readers, such as YTHDC1, YTHDC2, YTHDF1, YTHDF2, and HNRNPC; and erasers, specifically FTO and ALKBH5 [37]. As the most prevalent posttranscriptional modification of RNAs, m6A methylation plays a critical role in RNA metabolism at various levels, encompassing transcriptional and posttranscriptional regulation, RNA splicing, RNA stability, translation, and RNA localization [38]. Recent research has elucidated novel regulatory mechanisms involving circRNA‐m6A interactions [16], which are significant in cancer progression [27, 39]. For example, METTL3‐mediated m6A modification of circGLIS3 has been shown to promote prostate cancer progression and may serve as a potential target for androgen receptor signaling inhibitor (ARSI) therapy [40]. Additionally, circRNF13, a newly identified N6‐methyladenosine‐modified circular RNA, has been found to enhance radio‐resistance in cervical cancer by increasing CXCL1 mRNA stability [39]. The current study revealed that the regulation of circ_0006174 by METTL3 is dependent on m6A methylation, with the primary m6A methylation site identified at position 315.

In conclusion, our data demonstrated that circ_0006174 was upregulated in TNBC and was associated with poor prognosis in TNBC patients. The knockdown of circ_0006174 inhibited BC cell growth, invasion, and immune evasion. Mechanistically, circ_0006174 acted as a sponge for miR‐3139, thereby modulating PD‐L1 expression and influencing the progression of TNBC. Furthermore, circ_0006174 was regulated by METTL3‐dependent m6A methylation, with the primary m6A methylation site identified at position 315. Nonetheless, several limitations remain to be addressed in future studies. For instance, the role of circ_0006174 in other progresses of TNBC, such as cell motility, stemness, and drug resistance, warrants further investigation. Additionally, the direct effects of the circ_0006174/miR‐3139/PD‐L1 axis should be explored through in vivo experiments. Validation in immunocompetent or humanized models also represents a promising avenue for future research. In addition, one of the limitations of this study was the absence of a multivariable survival analysis that adjusts for significant confounders. Future research should conduct multivariate survival analyses on clinical cases to evaluate factors influencing prognosis and survival, such as age, pT stage, pTNM stage, chemotherapy, and adjuvant therapy. Furthermore, this study did not explore alternative pathways downstream of circ_0006174 or the initial stages of clinical validation. Briefly, this study was the first to identify circ_0006174 as a novel m6A‐modified circRNA and to validate the circ_0006174/miR‐3139/PD‐L1 axis as a potential therapeutic target for TNBC. Our research identifies a promising therapeutic candidate for the development of novel diagnostic strategies and therapeutic interventions in the management of TNBC, potentially advancing precision medicine approaches for this aggressive breast cancer subtype.

## Funding

This study was supported by the Honghu Program of International Peace Maternal and Child Health Hospital of China Welfare Society (HHJH2413, HHJH2415) and the Training Program for Discipline Leaders of International Peace Maternal and Child Health Hospital affiliated to Shanghai Jiaotong University (GFY9103).

## Conflicts of Interest

The authors declare no conflicts of interest.

## Supporting information


**Data S1:** Supporting Information.

## Data Availability

The data that support the findings of this study are available from the corresponding author upon reasonable request.
