# Supplementary material for: Circ_0006174 Drives Triple Negative Breast Cancer Progression and Immune Escape Through Regulating miR‐3139/PD‐L1 Axis
Source: Kaohsiung J Med Sci. 2025 Dec 24;42(6):e70156. doi: 10.1002/kjm2.70156 (PMC13248765; doi:10.1002/kjm2.70156)
Supplement: Supplementary file 1 — Data S1: Supporting Information. [file KJM2-42-e70156-s001.docx]

Supplementary Table 1 shRNA and miRNA sequences used in this study.

| Name | Sequences (5′-3′) |
| --- | --- |
| sh-circ_0006174#1 | GACAGGCAAAATCCTCAATGA |
| sh-circ_0006174#2 | GCAACTGACAGGCAAAATCCT |
| sh-circ_0006174#3 | GCATCCATCACTCCAGCATCA |
| sh-NC | TTCTCCGAACGTGTCACGT |
| miR-3139 mimic | UAGGAGCUCAACAGAUGCCUGUU |
| miR-NC | UCACAACCUCCUAGAAAGAGUAGA |
| anti-miR-3139 | CAGGCAUCUGUUGAGCUCCU |
| anti-NC | CAGUACUUUUGUGUAGUACAAA |
| si-METTL3 | GACTGCTCTTTCCTTAATA |
| si-NC | CCATTTACCCGAACGGCAA |


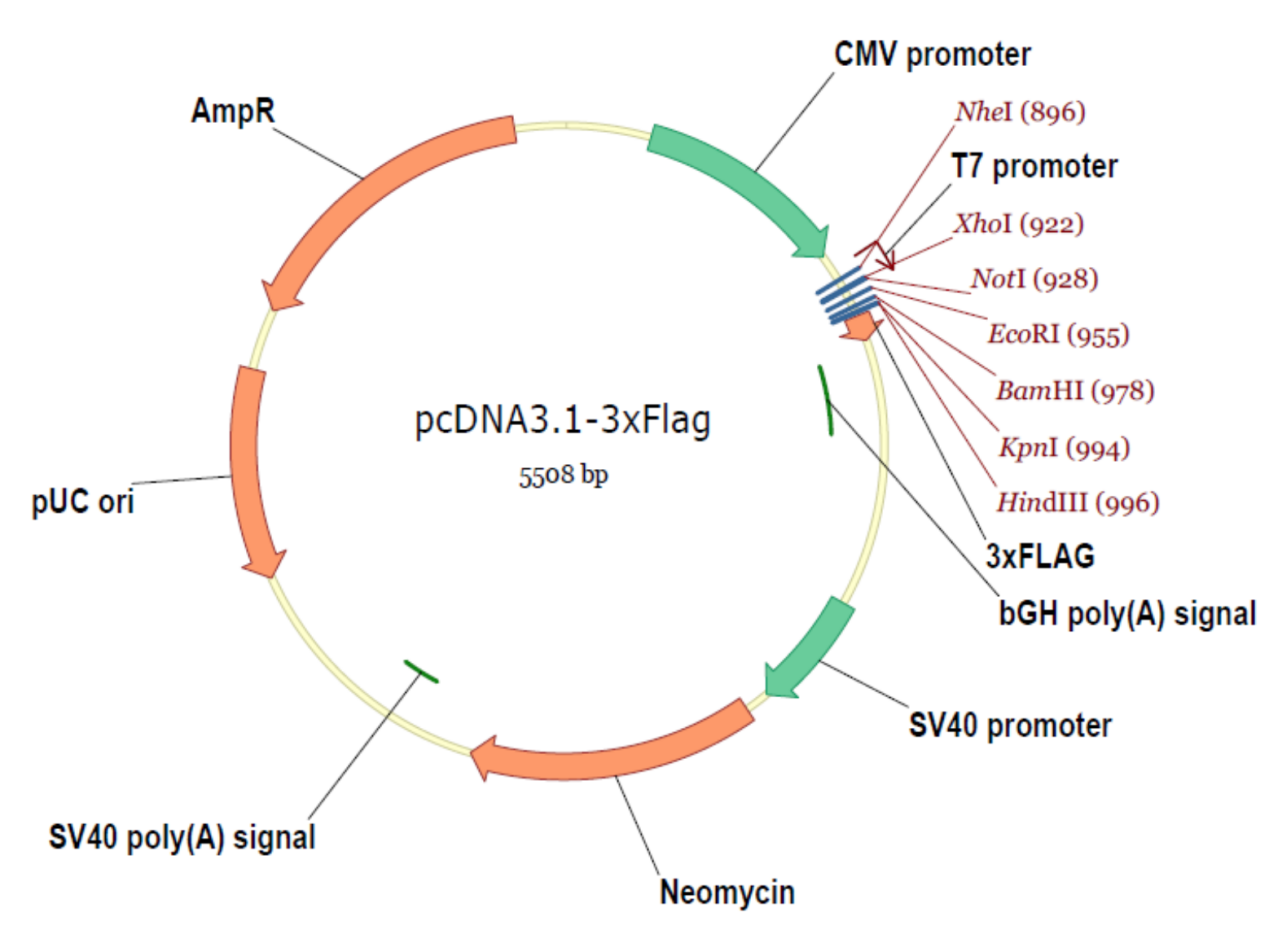


PD-L1

The inserted sequences of PD-L1:

AGTTCTGCGCAGCTTCCCGAGGCTCCGCACCAGCCGCGCTTCTGTCCGCCTGCAGGGCATTCCAGAAAGATGAGGATATTTGCTGTCTTTATATTCATGACCTACTGGCATTTGCTGAACGCCCCATACACAAAATCAACCAAAGAATTTTGGTTGTGGATCCAGTCACCTCTGAACATGAACTGACATGTCAGGCTGAGGGCTACCCCAAGGCCGAAGTCATCTGGACAAGCAGTGACCATCAAGTCCTGAGTGGTAAGACCACCACCACCAATTCCAAGAGAGAGGAGAAGCTTTTCAATGTGACCAGCACACTGAGAATCAACACAACAACTAATGAGATTTTCTACTGCACTTTTAGGAGATTAGATCCTGAGGAAAACCATACAGCTGAATTGGTCATCCCAGAACTACCTCTGGCACATCCTCCAAATGAAAGGACTCACTTGGTAATTCTGGGAGCCATCTTATTATGCCTTGGTGTAGCACTGACATTCATCTTCCGTTTAAGAAAAGGGAGAATGATGGATGTGAAAAAATGTGGCATCCAAGATACAAACTCAAAGAAGCAAAGTGATACACATTTGGAGGAGACGTAATCCAGCATTGGAACTTCTGATCTTCAAGCAGGGATTCTCAACCTGTGGTTTAGGGGTTCATCGGGGCTGAGCGTGACAAGAGGAAGGAATGGGCCCGTGGGATGCAGGCAATGTGGGACTTAAAAGGCCCAAGCACTGAAAATGGAACCTGGCGAAAGCAGAGGAGGAGAATGAAGAAAGATGGAGTCAAACAGGGAGCCTGGAGGGAGACCTTGATACTTTCAAATGCCTGAGGGGCTCATCGACGCCTGTGACAGGGAGAAAGGATACTTCTGAACAAGGAGCCTCCAAGCAAATCATCCATTGCTCATCCTAGGAAGACGGGTTGAGAATCCCTAATTTGAGGGTCAGTTCCTGCAGAAGTGCCCTTTGCCTCCACTCAATGCCTCAATTTGTTTTCTGCATGACTGAGAGTCTCAGTGTTGGAACGGGACAGTATTTATGTATGAGTTTTTCCTATTTATTTTGAGTCTGTGAGGTCTTCTTGTCATGTGAGTGTGGTTGTGAATGATTTCTTTTGAAGATATATTGTAGTAGATGTTACAATTTTGTCGCCAAACTAAACTTGCTGCTTAATGATTTGCTCACATCTAGTAAAACATGGAGTATTTGTAAGGTGCTTGGTCTCCTCTATAACTACAAGTATACATTGGAAGCATAAAGATCAAACCGTTGGTTGCATAGGATGTCACCTTTATTTAACCCATTAATACTCTGGTTGACCTAATCTTATTCTCAGACCTCAAGTGTCTGTGCAGTATCTGTTCCATTTAAATATCAGCTTTACAATTATGTGGTAGCCTACACACATAATCTCATTTCATCGCTGTAACCACCCTGTTGTGATAACCACTATTATTTTACCCATCGTACAGCTGAGGAAGCAAACAGATTAAGTAACTTGCCCAAACCAGTAAATAGCAGACCTCAGACTGCCACCCACTGTCCTTTTATAATACAATTTACAGCTATATTTTACTTTAAGCAATTCTTTTATTCAAAAACCATTTATTAAGTGCCCTTGCAATATCAATCGCTGTGCCAGGCATTGAATCTACAGATGTGAGCAAGACAAAGTACCTGTCCTCAAGGAGCTCATAGTATAATGAGGAGATTAACAAGAAAATGTATTATTACAATTTAGTCCAGTGTCATAGCATAAGGATGATGCGAGGGGAAAACCCGAGCAGTGTTGCCAAGAGGAGGAAATAGGCCAATGTGGTCTGGGACGGTTGGATATACTTAAACATCTTAATAATCAGAGTAATTTTCATTTACAAAGAGAGGTCGGTACTTAAAATAACCCTGAAAAATAACACTGGAATTCCTTTTCTAGCATTATATTTATTCCTGATTTGCCTTTGCCATATAATCTAATGCTTGTTTATATAGTGTCTGGTATTGTTTAACAGTTCTGTCTTTTCTATTTAAATGCCACTAAATTTTAAATTCATACCTTTCCATGATTCAAAATTCAAAAGATCCCATGGGAGATGGTTGGAAAATCTCCACTTCATCCTCCAAGCCATTCAAGTTTCCTTTCCAGAAGCAACTGCTACTGCCTTTCATTCATATGTTCTTCTAAAGATAGTCTACATTTGGAAATGTATGTTAAAAGCACGTATTTTTAAAATTTTTTTCCTAAATAGTAACACATTGTATGTCTGCTGTGTACTTTGCTATTTTTATTTATTTTAGTGTTTCTTATATAGCAGATGGAATGAATTTGAAGTTCCCAGGGCTGAGGATCCATGCCTTCTTTGTTTCTAAGTTATCTTTCCCATAGCTTTTCATTATCTTTCATATGATCCAGTATATGTTAAATATGTCCTACATATACATTTAGACAACCACCATTTGTTAAGTATTTGCTCTAGGACAGAGTTTGGATTTGTTTATGTTTGCTCAAAAGGAGACCCATGGGCTCTCCAGGGTGCACTGAGTCAATCTAGTCCTAAAAAGCAATCTTATTATTAACTCTGTATGACAGAATCATGTCTGGAACTTTTGTTTTCTGCTTTCTGTCAAGTATAAACTTCACTTTGATGCTGTACTTGCAAAATCACATTTTCTTTCTGGAAATTCCGGCAGTGTACCTTGACTGCTAGCTACCCTGTGCCAGAAAAGCCTCATTCGTTGTGCTTGAACCCTTGAATGCCACCAGCTGTCATCACTACACAGCCCTCCTAAGAGGCTTCCTGGAGGTTTCGAGATTCAGATGCCCTGGGAGATCCCAGAGTTTCCTTTCCCTCTTGGCCATATTCTGGTGTCAATGACAAGGAGTACCTTGGCTTTGCCACATGTCAAGGCTGAAGAAACAGTGTCTCCAACAGAGCTCCTTGTGTTATCTGTTTGTACATGTGCATTTGTACAGTAATTGGTGTGACAGTGTTCTTTGTGTGAATTACAGGCAAGAATTGTGGCTGAGCAAGGCACATAGTCTACTCAGTCTATTCCTAAGTCCTAACTCCTCCTTGTGGTGTTGGATTTGTAAGGCACTTTATCCCTTTTGTCTCATGTTTCATCGTAAATGGCATAGGCAGAGATGATACCTAATTCTGCATTTGATTGTCACTTTTTGTACCTGCATTAATTTAATAAAATATTCTTATTTATTTTGTTACTTGGTACACCAGCATGTCCATTTTCT TGTTTATTTTGTGTTTAATAAAATGTTCAGTTTAACATCCCA


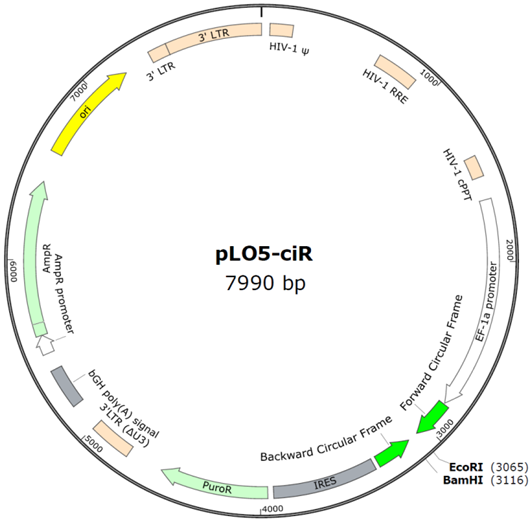


circ_0006174

The inserted sequences of circ_0006174:

GCAAAATCCTCAATGATGATACTGCTCTCAAAGAATATAAAATTGATGAGAAAAACTTTGTGGTGGTTATGGTGACCAAAGTAAGTTTCAACCTCATTCTGTATATCTTTATGCATGTAGGTCTTTTTAAAAATGATGATCACAAGTCCACACAATGGACACAGTTTATACACATCCATAGTGGTGTACACTTAACTACTATGAAAGGTTTTTAAGAATTTACCTTAAGTGAAACATAAAGTTATTAATGTTTTACATTAATTACACATTAAACTATAATATAGTTTTAATAAAATTTTATGTAAATTAAAACAATTGAAAAATAAAATGTCATGTTAAAATTATTTTTGCTACAGTAGTGAAATCATTATATAATTCTTTTAAAATGTCTTTATAAGTGGAATAAATGTTTATTCTGTAGTTTTTGTAAGCTTGCCTTAAATAGACCTGCTAAATTACGTACTAGTCCTAACAGATTCCTCAAGACAGAAAAGGCATGAAAGCTTATTATTACAGTGATAGTTCTTAGTGAAAGAATATGTTTTTAGAAACTAAAAAAAGGGAAAAGATAACTAAAGAAATGATGGAATGATTTTTATGAGGAAGTTTTTTTTGTTTTTGTTTTGTTTTTTTTTTTTTGAGACGGAGTCTCGCTCTGTTGCCCAGGCTGGAGCGCAGTGACGCTGTCTTGGCTCACTACAAGCTCTGTCTCCCGGGTTCATGCCATTCTCCTGCCTCAGCCTCCCTAGTAGCTGGGACTACAGGCGCATGCCACCACGCCCGGCTAATTTTTTTGTATTTTTAGTAGAGACAGGGTTTCACCGTGTTAGCCAGGATGGTCTCGATTTCCTGACCTCGTGATCCGCCCGCCTCGGCCTCCCAAAGTGCTGGGATTACAGGCGTGAGCCACCGCGCCCAGCCTGGAAGTTTTAAACAGATACTTTTTTGTGATTTAGTAGGCTGGAATGTATTTGGAACAATTTATAAAATTAAATTTTTTTTAAAGCAGAGAATGAACATGGATTTTTTTAAGAGACTATTTATATACTTGTTTATTTAAAGATATATAATGCAGTGTAGAATTAAGAGACAAATGGAGAAGTGGTACTCATGACATTCATTTTTAGGATTGGATTGTCATAATCTGAGAAAGAACAGTGTTACTTGTAGCACTGAAAATGAGTTTAAATCACTAATTGAAACCACTTCATCAAAGGCCAAAAGTGGATTAAATGTGGGTTTTTTCTCAAAGTCACAAAATAGTTAATAGTAATAGAAAATAGAGTCGAAGAAATAAATTCTAATTACCACATGCTTTTGTACTGGGAGCAAGCAGCTAAATAGTTTACAAGTGTTGAACATTTTTTAATTCATTAAGAACCATGTTGATTTTGCTATACTTAAGAACTTAAGTCATTATATGTTAAGTTTAAAAACAAGCAGAATCGTGAGGAAACCTTGTATCATCTAGGTTTTTTTATTCAACTGTGGTAAAATATAACAAACTTCCAGTTTAATCATTTTAAGTGTACAGTTCAATAACATTATAGTTACATTGTTTTGCAGCTATCACCATCATATAAGTGGAATCCTATGGTATTTGTCTTTTTGTGACTGGCTTATTTCACTTAGCATAATGTCATTGAGGTTCATCTGTCTATGTTATAGCTTGTGCCAGAATTTGCTGAACTTTTAAGACTGAATACTATCCTATTTTCCTATCTAGTTTTAGCATACATAGTACATAATGGGAAATGTGATAGCCACACATTTAAATATGTTTTGTTACTGGTCTTAGTTTCTTTTAATATTACAGTGATCCTGACTGTTTAGAACACAAGAATTAAGCAGAAAAAGTCTCAATAGGTGCGTGTGTTTGAGGAATAGATACTTGAAGCCTATCATTATATTGCATACAGTCCTTTTGTTTTTCCTTAAAGTCTTTTATTCCTAATATTACATAGGATTCTAATGATATTTATGATTGTTTTATTTGAAGTTAATAGAGGACAACTATGAAAAGGGAGGGAATGCTGCTACAGTAAGGCATTATGGAAGCACTGAGAAATGACCTTATTAATACAAAGAAGGGAAAGGAAAAAGCTATTAAGATCAAAAGCAAGAATAAAAACTAAAAAGCAGAGAAGGGCTTGGTACTGTTTAGAATAAAGTATAAGTAGCTGTTCTTGGCAGTAGGAACCTTGAAGGGAAGTTACATTGCAATATTATTTTGATAACAATTAAGGAAAATATTAAATGAGTATACCACTTTTAAAAATACATTTTTATCTAGATTGGTTATTATAAAGTTTTGGTGCTTAAGACTCTAAATTTTCACCTTTGTGAAGCTCCAGTTTATCCAGAATAACTGAAGATCCAGAATGATCTCAGGATTTCAGCTCATTTTAGTTTTAGAACAGTAATAATCTGCAGAATATTATATTGGCTGTTAAGAAAGTTAAGCTAAATAGGAAAAGAAGGGAGTAATGCTACTAGTATTTTTTAAAAACCTATCACCTTTCACTAACCTTTTTCCTAGTGTCTTCTTTATTAGTTCAAATCAAAAAGACAAGGCAGCTCATTGGTTTTAGAGTTTGTTTATGCCTGTGGCAAATTATGTTGAATTTAAGCCTTGTCTTCCACTAAAACAGCTACCTTTTGTCCTTGGGAAAGTTATGAACTAAACAGGTAGCGAGCATTGAGAGGCCTTAGGAGAAGACTAAAAATCATGTATGATTTTTACACATGTACAGTTGACCCTTGAACACCACTGAGCTTAGGAGTGCTGGCCTCTCCCCTACAGTCAGAAATCTGCATATACCTTTTGACTTGCCCAAAACTTACCTACTAATAGCCTACTCTTGACCAGAAGCCTTACTGATAACGTAAACAGTGGATTAATACATGTTTTATATGTTACATGTATGATATACTGTATTAAAGTAAGCTAGAGAAACTATTAAGAAAATCATGGCCAGGTGTGGTGGCTGATGCCTGTAATCCCATCGCCTTGGGAGGCCAAGGTGGGCGGATCGCTTGAGCTCAGGTGTTGGAGACCTGTCTGGGTAACATGGCGAAACCTTGTCTCTACAAAAAAAAAAAATACAAAAATTTAGCAGGGAGTGGTAGTGTGAGCCCGTAGTCCCAGCTACCTGGTGTTGGGGGCGGGGGCAGTGGCTGAGGCAGGAGCCTTGGGAGGTTGAGGCTGCAGTGAGCTGTGATCATACCACTGCACTCCAGTCTGGGCGATAGAGTGAGAACCTCTCTCAAAAAGAAACAAAAGAAAATCACGAGAGAGAATATTTTTAGTGTTCATTAAGTGTAAGTGGATCATCATAAAGGTCTTCATCCTTGTCTTCACACTGAATAGGCTGAGAAAGAGAAAGGGTTGGTATTGCGGTCTCAAGGTGGCAGAGATGGAGGAGGTAGAAGGAGAGGCAGGCACACTCAGTGTAACTTATATTGAAAAAAATTATCATGTAAGTGGACCCAGTGCAGTTCAAACCCTTGTTGTTCAAGGGTCAACTAATTGTTTTTTTGCTCTGTGGGAAGCTTTATGGCTCTATTTCCAGTTCAGAAAGTGCTGAGCGTGTGGAGGTAACTTAATTTGGCCTTTATTTTTCTGCTTAATTACATATATGTCTGAAAGTATTGTGAACCTTTTTGTACTTGGATCTGTTGATTTGTAGTCAATTTTGGGATTTTCCTCCCTCTTCTAAAAATACAGTACAACAGAATATTTGACATGTTTATAAATAAACATTTATACTGCCATTTCAAGAACATTATTTTGGGCTGGATGTAGTAGTTTATGCCTGTAATCCTAGCGCTTTGGGAGCCCGTGGTGGGAGGATTGCTTCAGGCCCGGAGTTCAAGACCAGCCTGGGCAAGGTAGCAAGACCCTGTCTTTAAAATTAAAGAAAATTATTTTATTAATGCTTTTAAGTGGTATTAGGTACCTTAAAGTTACAACTTAGGTACATAAAGTTTTTTTGGGAAAATGATACGGTTTATATCTATATATATATATATATATATCTATATATCTATATATATTTCAAATTTTTTATTTTATATTAACTTGATATGAAGATGGAAATTGTTGGGTAAAATTAATTGTGGTTGATAGTTAAGATTAACATACGCTGTCATGAGCTTTGAAAGAGTAACCATGAGTCAGATTGTTAATATTGATCATTTATTGTGTCATTTTACAAAATTATTTTCAAGTTATAGGTTGTTATTAGTGCTATGTTTGTGGCATCCTGTAACGGTACAGTCTAATTAGAGATCAGGCAGTATGTAGTATTTTATTGTAATTATTTTGTCTTTTAATGTGTTTAGCCCAAAGCAGTGTCCACACCAGCACCAGCTACAACTCAGCAGTCAGCTCCTGCCAGCACTACAGCAGTTACTTCCTCCACCACCACAACTGTGGCTCAGGCTCCAACCCCTGTCCCTGCCTTGGCCCCCACTTCCACACCTGCATCCATCACTCCAGCATCAGCGACAGCATCTTCTGAACCTGCACCTGCTAGTGCAGCTAAACAAGAGAAGCCTGCAGAAAAGCCAGCAGAGACACCAGTGGCTACTAGCCCAACAGCAACTGACAG
